# Recombinant Production, NMR Solution Structure, and Membrane Interaction of the Phα1β Toxin, a TRPA1 Modulator from the Brazilian Armed Spider *Phoneutria nigriventer*

**DOI:** 10.3390/toxins15060378

**Published:** 2023-06-03

**Authors:** Ekaterina N. Lyukmanova, Pavel A. Mironov, Dmitrii S. Kulbatskii, Mikhail A. Shulepko, Alexander S. Paramonov, Elizaveta M. Chernaya, Yulia A. Logashina, Yaroslav A. Andreev, Mikhail P. Kirpichnikov, Zakhar O. Shenkarev

**Affiliations:** 1 Department of Biology, MSU-BIT Shenzhen University, Shenzhen 518172, China; 2 Shemyakin-Ovchinnikov Institute of Bioorganic Chemistry, Russian Academy of Sciences, 119997 Moscow, Russia; 3 Phystech School of Biological and Medical Physics, Moscow Institute of Physics and Technology (State University), 141701 Dolgoprudny, Russia; 4 Interdisciplinary Scientific and Educational School of Moscow University “Molecular Technologies of the Living Systems and Synthetic Biology”, Faculty of Biology, Lomonosov Moscow State University, 119234 Moscow, Russia; 5 National Research University Higher School of Economics, 101000 Moscow, Russia; 6 Institute of Molecular Medicine, Sechenov First Moscow State Medical University, 119991 Moscow, Russia; 7 International Tomography Center SB RAS, 630090 Novosibirsk, Russia

**Keywords:** NMR, disulfide bond pattern, knottin peptide, spider toxin, TRPA1

## Abstract

Phα1β (PnTx3–6) is a neurotoxin from the spider *Phoneutria nigriventer* venom, originally identified as an antagonist of two ion channels involved in nociception: N-type voltage-gated calcium channel (Ca_V_2.2) and TRPA1. In animal models, Phα1β administration reduces both acute and chronic pain. Here, we report the efficient bacterial expression system for the recombinant production of Phα1β and its ^15^N-labeled analogue. Spatial structure and dynamics of Phα1β were determined via NMR spectroscopy. The *N*-terminal domain (Ala1–Ala40) contains the inhibitor cystine knot (ICK or knottin) motif, which is common to spider neurotoxins. The *C*-terminal α-helix (Asn41–Cys52) stapled to ICK by two disulfides exhibits the µs–ms time-scale fluctuations. The Phα1β structure with the disulfide bond patterns Cys1–5, Cys2–7, Cys3–12, Cys4–10, Cys6–11, Cys8–9 is the first spider knottin with six disulfide bridges in one ICK domain, and is a good reference to other toxins from the ctenitoxin family. Phα1β has a large hydrophobic region on its surface and demonstrates a moderate affinity for partially anionic lipid vesicles at low salt conditions. Surprisingly, 10 µM Phα1β significantly increases the amplitude of diclofenac-evoked currents and does not affect the allyl isothiocyanate (AITC)-evoked currents through the rat TRPA1 channel expressed in *Xenopus* oocytes. Targeting several unrelated ion channels, membrane binding, and the modulation of TRPA1 channel activity allow for considering Phα1β as a gating modifier toxin, probably interacting with S1–S4 gating domains from a membrane-bound state.

## 1. Introduction

**Figure 1:**
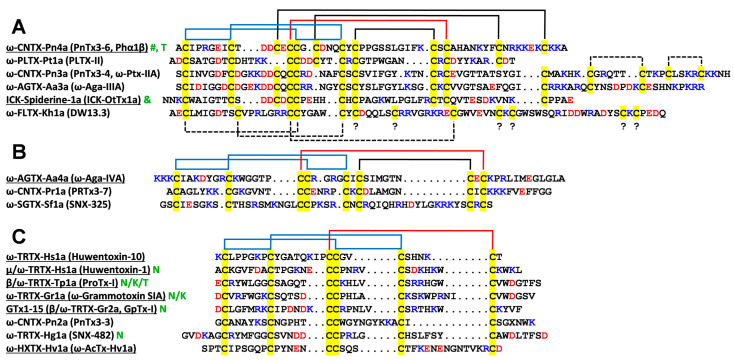
Sequence alignment of Phα1β (ω-CNTX-Pn4a, marked with green symbol “#”) and spider toxins targeting calcium (Ca^2+^) channels. Toxins containing five and more disulfide bonds (**A**), four disulfide bonds (**B**), and three disulfide bonds (**C**) are shown. Toxins with known spatial structures are underlined. The ICK domain of Spiderine-1a from *Oxyopes takobius* (marked with green symbol “&”) does not target Ca^2+^ channels, but represents a spider knottin with five disulfide bridges, with a known spatial structure. Solid blue and red lines show the ICK motif. The blue lines show the loop formed by the backbone fragments and two disulfides. The red line shows the third disulfide passing through this loop. Solid lines indicate experimentally determined disulfide bonds, and dashed lines indicate the predicted disulfide bonds. Cys residues are highlighted by yellow. The sign “?” denotes Cys residues with uncertain pairings. Positively and negatively charged residues are colored by blue and red, respectively. The green symbols “T”, “N”, and “K” denote toxins that additionally target TRPA1, Na^+^, or K^+^ channels, respectively.

Venoms of animals are rich sources of selective ligands to various receptors and ion channels [1,2]. These ligands (toxins) can be used for the biochemical characterization of the target receptors and serve as prototypes for new drugs and insecticides [2,3,4]. For example, some ion channel blockers found in spider venom show promising antinociceptive properties and could be considered potential analgesics [5,6]. Phα1β toxin (also known as PnTx3-6) is a 55 a.a. peptide isolated from the venom of the Brazilian armed spider, *Phoneutria nigriventer* (Figure 1A) [7,8]. Phα1β exhibits analgesic properties and, in animal pain models, reduces both acute and chronic pain [9,10,11,12]. Initially, Phα1β was characterized as a selective inhibitor of the high-voltage-activated calcium channels from the Ca_V_2 subfamily [13,14]. The toxin completely blocked currents through Ca_V_2.2 (N-type) and partially blocked the Ca_V_2.1 and Ca_V_2.3 channels (P/Q- and R-types, respectively) [14]. Later, the antagonistic action of Phα1β at the transient receptor potential ankyrin 1 (TRPA1) ion channel was described [15]. The Phα1β effect was selective for the TRPA1 channel; the toxin did not affect calcium responses evoked by the activation of TRPV1 and TRPV4 channels [15].

TRPA1 is a homotetrameric, non-selective cation channel with a high permeability for calcium ions [16,17,18,19]. TRPA1 substantially contributes to human chemical and temperature sensitivity, and is activated by noxious cold and various exogenic and endogenic compounds which can elicit pain. The TRPA1 channel contributes not only to acute pain sensation and the development of the inflammation, but may also be involved in transition from acute to chronic pain [20,21]. TRPA1 inhibition is one of the promising strategies for neuropathic pain therapy. Phα1β studies are very relevant in this regard; presently, only a limited number of natural compounds that inhibit the TRPA1 channel are known [6,17].

Phα1β demonstrates a more prolonged antinociceptive effect and causes fewer adverse reactions compared to the classic Ca_V_2.2 blocker ω-conotoxin MVIIA, which was introduced into the clinic as an atypical analgesic under the name Ziconotide [11,12,22]. Most probably, the stronger antinociceptive effect of Phα1β is due to its dual action, the simultaneous block of the Ca_V_2.2 and TRPA1 channels [12]. For the N-type Ca^2+^ channels, it was proposed that Phα1β binds to the outer mouth of the channel and physically occludes the pore, blocking the calcium influx [14]. However, another mechanism is probably responsible for the partial blockade observed on the P/Q- and R-types of Ca^2+^ channels [14]. The mechanism of the Phα1β action on the TRPA1 channel and the localization of the toxin-binding site are currently unknown. At the same time, the distantly related tarantula toxin, ProTx-I (Figure 1C), blocks the TRPA1 channel by binding to the S1–S2 loop of the S1–S4 gating domain [23]. Thus, modification of the channel gating is the possible mechanism of the Phα1β action on TRPA1.

Spider toxins that affect the gating of the channels from the ‘P-loop’ superfamily (which includes voltage-gated Na^+^, K^+^, and Ca^2+^ channels, as well as TRP channels) typically target the S1–S4 gating domains [24]. These domains (four in a full channel) are embedded in the membrane and usually have short extracellular loops. Therefore, some spider toxins follow a special tactic: they attack the S1–S4 domains from the membrane surface [25,26,27]. These so-called “voltage-sensor toxins”, including ProTx-I, have a large hydrophobic region on their surfaces and exhibit an affinity for lipid membranes [27,28,29].

The gating modifier compounds that target the S1–S4 domains often exhibit promiscuous interactions with different types of ion channels. For example, many tarantula toxins demonstrate simultaneous activity against several subtypes of Na^+^ and K^+^ channels [30]. This also applies to the toxins which inhibit Ca^2+^ channels; many of them are also active against Na^+^ and K^+^ channels (Figure 1, green symbols). A notorious example is ProTx-I, which interacts not only with the TRPA1 channel [23], but also with several subtypes of Na^+^, K^+^, and Ca^2+^ channels [31]. There are also examples of low-molecular-weight gating modifiers that simultaneously target the S1–S4 domains in the H^+^, K^+^, and TRPV1 channels [32].

The spatial structure and connectivity of disulfide bonds in the Phα1β molecule have not been established. Homologues toxins from spider venoms belong to the knottin peptide family and contain an inhibitory cysteine knot (ICK) motif that includes three SS-bonds with the Cys1–4, Cys2–5, Cys3–6 arrangement (Figure 1 blue and red lines). Although, the structures of the spider knottins with up to five disulfide bridges (Spiderine-1a [33], Figure 1A) are known, the Phα1β molecule probably contains six SS bonds, and their connectivity pattern remains uncertain. In addition, the recent mass spectrometry study of a recombinant Phα1β analogue confirmed the formation of all six disulfide bonds, but the identified disulfide bond arrangement (Cys1–2, Cys3–4, Cys5–6, Cys7–8, Cys9–10, and Cys11–12) was inconsistent with the ICK topology [34].

Here, we report the recombinant production, structural and dynamic study, and electrophysiological characterization of Phα1β at the rat TRPA1 channel expressed in *Xenopus laevis* oocytes. The NMR investigation of the ^15^N-labeled toxin revealed the Phα1β spatial structure containing the ICK motif with the disulfide bond patterns Cys1–5, Cys2–7, Cys3–12, Cys4–10, Cys6–11, Cys8–9, and discovered the presence of the intramolecular µs–ms time-scale motions associated with the fluctuation of the *C*-terminal helix (Asn41–Cys52). Lipid titration experiments revealed a moderate affinity of the toxin for negatively charged lipid vesicles under low-salt conditions. Contrary to expectations, 10 µM Phα1β significantly (up to ~150–200%) increased the amplitude of ion currents through the TRPA1 channel activated by the non-covalent agonist diclofenac and did not affect the amplitude of ion currents evoked by the covalent agonist allyl isothiocyanate (AITC). The determination of the exact 3D structure and improved understanding of the toxin pharmacology could greatly assist future pharmacological development of next-generation antinociceptive drugs.

## 2. Results and Discussion

### 2.1. Expression and Purification

The structure–function studies of Phα1β require large quantities of the toxin and its mutants. Phα1β is a rather small protein (55 a.a.) and contains six disulfide bonds, so its recombinant production as a solo protein in the correctly folded form seems to be a difficult task. To prevent the formation of non-native disulfide bonds and subsequent aggregation of the toxin due to misfolding, we designed a construction where Phα1β is expressed as a fusion protein with thioredoxin (TRX). TRX is a known partner for the production of disulfide-rich proteins, and can increase the yield and stability of correctly folded recombinant proteins [28,35,36]. To further promote the folding of Phα1β, we used the SHuffle *E. coli* strain and decreased the temperature of cell cultivation after induction to 13 °C. This approach allowed us to significantly increase the expression of the TRX–Phα1β fusion protein in the soluble cytoplasmic fraction.

**Figure 2:**
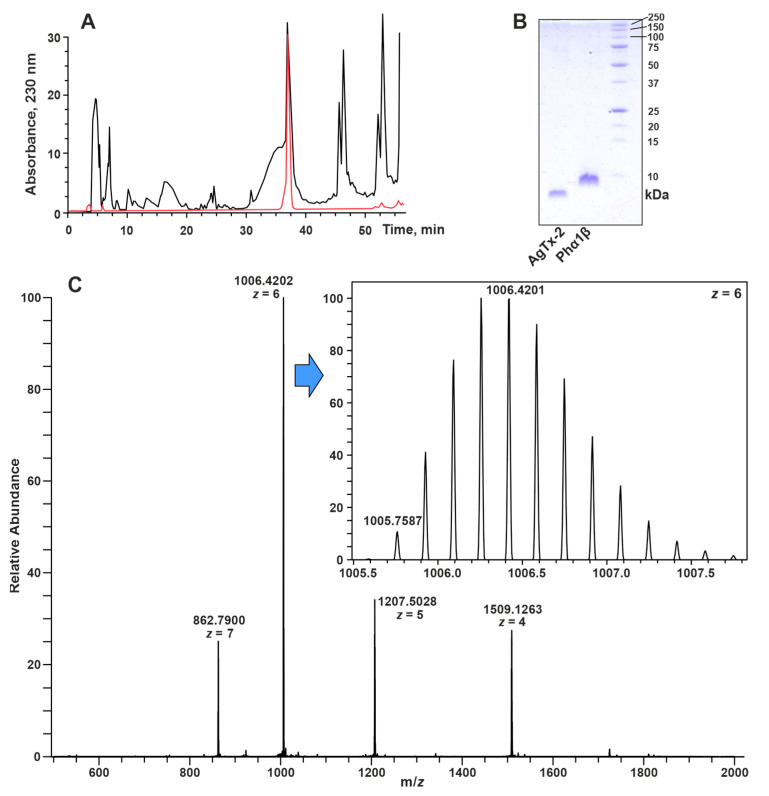
Characterization of the recombinant Phα1β protein. (**A**) Representative HPLC chromatograms of Phα1β purification. Chromatogram of the TRX–Phα1β fusion protein cleaved by BrCN is shown in black and the analytical HPLC of the purified Phα1β is in red. (**B**) SDS-PAGE analysis of the purified Phα1β (MW~6 KDa). Agiotoxin-2 (MW~4 Kda) was used as a low-molecular-weight reference protein. The full SDS-PAGE gel is shown in Figure S1. (**C**) MS spectrum of the fully oxidized recombinant Phα1β protein (expected monoisotopic mass of [M+H6]^6+^ ion is 1005.755 Da). The spectrum was obtained via LC-MS using Q Exactive Plus Orbitrap mass spectrometer (Thermo Fisher Scientific, Waltham, MA, USA). The full LC-MS profile is shown in Figure S2.

The TRX–Phα1β fusion protein was purified on Ni–Sepharose using the His6-tag in the linker connecting the two proteins. The fusion protein was cleaved with BrCN at the Met residue presented in the linker just before the toxin sequence. The final purification of Phα1β was carried out using reverse-phase HPLC (Figure 2A). The identity and purity of the obtained recombinant toxin was confirmed by HPLC, SDS-PAGE and LC-MS analyses (Figure 2). The mass-spectrometry analysis confirmed that all 12 cysteines in the recombinant Phα1β toxin form disulfide bonds. The observed monoisotopic mass 1005.759 Da of the [M+H6]^6+^ ion nicely corresponds to the theoretically calculated mass 1005.755 Da of the Phα1β molecule minus 12 ^1^H atoms (Figure 2C).

The yield of the isolated Phα1β was 14 mg and 0.6 mg per liter of bacterial culture for unlabeled and ^15^N-labeled toxins, respectively. The expression on M9 minimal medium used for production of isotopically labeled proteins is usually less efficient than the expression on TB reach medium used for production of unlabeled proteins. Typically, we get a 5-fold drop in the expression yield. However, here the yield of the labeled protein was more than 20 times lower than the yield of the unlabeled one. The conditions found for the protein expression in the rich TB medium (cell density upon induction, temperature, speed of rotation, time of cultivation) were likely not optimal for the expression on the minimal medium. Another reason may be the membrane activity of Phα1β (see below), which did not greatly affect cell growth in the TB medium, but became a decisive factor for cell growth under stress conditions (minimal medium).

### 2.2. Spatial Structure of Phα1β

**Figure 3:**
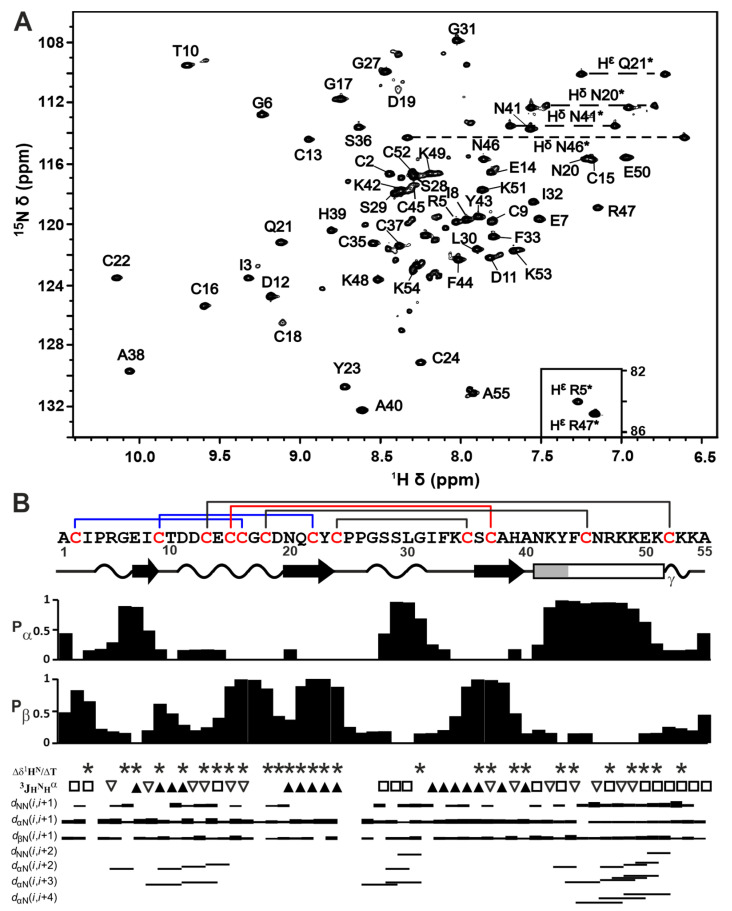
NMR data define the Phα1β structure in solution. (**A**) 2D ^15^N-HSQC NMR spectrum of ^15^N-Phα1β (30 °C, pH 4.5). The resonances of the side-chain NH and NH_2_ groups are marked by an asterisk. (**B**) Secondary structure of Phα1β. The elements of the secondary structure in the determined spatial structure of the toxin were calculated using the STRIDE program [40]. The β-strands are designated by black arrows, α-helix by rectangle (probable region of the 3_10_ helix is gray), and tight β/γ-turns by wavy lines. Cys residues are colored in red. Pα and Pβ are the probabilities of the residue involvement in the α-helix or β-strand, respectively, obtained from the analysis of chemical shifts in the TALOS-N software [39]. The residues, of which the temperature gradients of the amide proton (Δδ^1^H^N^/ΔT) are lower than 4.5 ppb/K in magnitude, are designated with an asterisks. The small (<6 Hz), large (>8 Hz), and medium (others) ^3^J_H_^N^_H_^α^ coupling constants are designated by empty triangles, filled triangles, and open squares, respectively. The map of NOE contacts (τm = 100 ms) is shown, as usual. A scheme of the disulfide bonds is shown above the amino acid sequence. Solid blue and red lines show disulfide bond connectivity in the ICK motif (see the legend of Figure 1).

The spatial structure of Phα1β was studied via NMR spectroscopy in an aqueous solution using unlabeled and ^15^N-labeled toxin samples (Figure S3). The measured 2D ^15^N-HSQC spectrum (Figure 3A) showed a high resonance dispersion characteristic of the folded β-structural proteins. At the same time, some of the backbone HN resonances were significantly broadened and had a low intensity (e.g., Cys18, Asp19, and Cys45, Figure 3A). To reduce the signal-broadening caused by the exchange of the HN protons with the solvent (water), we performed a structural study at a moderately acidic pH. Although minimal solvent exchange rates are typically observed at pH ~3, we used pH = 4.5 to keep the ionization state of the acidic groups (Asp, Glu side chains, and C-terminal carboxyl) closer to physiological (deprotonated and negatively charged). The pKa values of these groups in the blocked tripeptides are 3.86, 4.34, and 3.55, respectively [37]. At the same time, the His sidechain group at this pH is probably protonated and uncharged (pKa of 6.45).

The signals of all the Phα1β residues were observed in NMR spectra measured at pH 4.5 and 30 °C. At the same time, an additional set of weak resonances was present in the ^15^N-HSQC spectrum (Figure 3A). Considering the purity of the Phα1β sample confirmed by HPLC, SDS-PAGE, and LC-MS (Figure 2) analyses, we can propose that these additional signals belong to the disulfide isomer of the peptide or the peptide conformer with cis–trans isomerism of Xxx–Pro peptide bond(s). Indeed, the LC-MS analysis of the Phα1β sample revealed the presence of two chromatographically distinct isomers with the same set of m/z values (Figure S2). Using a combination of the 2D/3D TOCSY and NOESY spectra (Figure S4) and the 2D ^13^C-HSQC spectrum measured at a natural isotopic abundance (Figure S5), an almost complete assignment of the ^1^H,^15^N resonances and a partial assignment of the ^13^C resonances of the major peptide isoform were obtained.

The difference in the chemical shift δ[^13^C^β^]–δ[^13^C^γ^] (Δ_βγ_) for the proline residues measured in the ^13^C-HSQC spectrum (Figure S6) made it possible to determine the configuration of the Xxx–Pro peptide bonds in the major isoform of Phα1β. The Δ_βγ_ values for Pro4, Pro25, and Pro26 (4.51, 3.65, and 5.84 ppm, respectively) were in the range 4.51 ± 1.17 ppm, typical for trans-Xxx–Pro dipeptides [38]. The presence of strong H^α^_i_–H^δ^_i+1_ cross-peaks (Figure S8) and the absence of the H^α^_i_–H^α^_i+1_ contacts for the Xxx–Pro dipeptides in the NOESY spectrum also confirms the trans configuration. No additional Pro spin systems were observed in the TOCSY (Figure S7) and ^13^C-HSQC spectra (Figure S6), indicating the absence of the cis–trans isomerization of the Xxx–Pro bonds in the Phα1β. Thus, the additional set of signals observed in the ^15^N-HSQC spectrum (Figure 3A) probably corresponds to the disulfide isomer.

The analysis of the backbone chemical shifts in the TALOS-N software [39], together with the measured ^3^J_H_^N^_H_^α^ coupling constants and NOE connectivities (Figure 3B), provided information about the secondary structure of Phα1β. According to these data, the long helix is formed in the Asn41–Lys51 region. The distribution of the H^α^_j_–H^N^_j+4_ NOE contacts suggested that the *N*-terminal fragment of this helix (Asn41–Phe44) can adopt the 3_10_ conformation, while the *C*-terminal region forms an α-helix (Figure 3B). The α-helical conformation of the short Arg5–Ile8 and Ser28–Ile32 regions was not supported by the ^3^J_H_^N^_H_^α^ data, indicating that these fragments rather form the β-turns or isolated turns of the 3_10_ helix. Similarly, all NMR data supported the formation of the β-strands in the Asn20–Cys24 and Cys35–His39 regions, while the β-structural conformation of the Cys15–Cys19 fragment was not supported by the ^3^J_H_^N^_H_^α^ values (Figure 3B).

**Figure 4:**
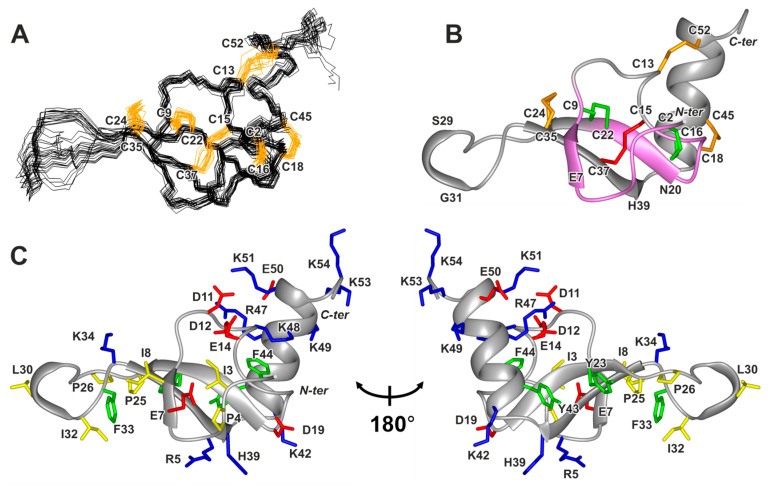
Spatial structure of Phα1β in solution. (**A**) Set of the 20 best CYANA structures. All disulfides are shown in orange. (**B**) Representative conformer in ribbon representation. The fragments of the backbone and disulfide bonds forming the Cys knot are colored. The loop formed by the backbone fragments and two disulfides is highlighted in violet and green. The third disulfide passing through this loop is shown in red. Other disulfide bonds are shown in orange. (**C**) Two-sided view of the Phα1β molecule. Positively charged (including His), negatively charged, hydrophobic, and aromatic residues are colored blue, red, yellow, and green, respectively.

The set of 20 Phα1β structures (Figure 4A) was calculated in CYANA [41] from 200 random starts, using the following experimental data: upper NOE-based distance restraints, J coupling-based torsion angle restraints for φ and χ^1^ angles, disulfide bond restrains, and hydrogen bond restraints (the HN-groups with |Δδ^1^H^N^/ΔT| < 4.5 ppb/K were assumed to be hydrogen-bonded) (Table S1). The following calculation protocol was used to determine the disulfide bond pattern. The analysis of the 2D NOESY spectra revealed the presence of H^β^–H^α^ NOE contacts for the Cys2–Cys16 and Cys24–Cys35 pairs, which confirms their disulfide connectivity. We then calculated the preliminary Phα1β structure based only on these two disulfide bonds. An analysis of pairwise S–S distances in the calculated set of structures additionally suggested the closure of the Cys9–Cys22 and Cys15–Cys37 bonds. Setting restraints for the Cys2–Cys16, Cys24–Cys35, Cys9–Cys22, and Cys15–Cys37 bonds in the next round of the structure calculation resulted in a significant decrease in the CYANA target function and an increase in structural convergence. This disulfide pattern corresponds to the conventional ICK fold, with one additional disulfide (Cys24–Cys35, Figure 4B).

The preliminary set of structures represented the compact ICK core with the elongated Cys24–Cys35 loop and protruding *C*-terminal helix (Asn41–Lys51). No NOE contacts were observed between this helix and the ICK part of the molecule, probably due to dynamic fluctuations in the helix (see below). At the same time, the remaining cysteines can be closed only in the Cys18–Cys45/Cys13–Cys52 arrangement. These disulfides restrict the position of the *C*-terminal helix relative to the ICK motif. The resulting disulfide bond arrangement is also supported by the observed broadening of the Cys18 and Cys45 resonances, indicating their involvement in the same (or similar) µs–ms time-scale exchange process(es). Interestingly, the calculated Phα1β structure and disulfide connectivity are consistent with the model predicted by AlphaFold 2.0 [42] (Figure S9). The minimal RMSD values between the experimentally determined set of structures and the model are only 2.5 Å (backbone) and 3.4 Å (heavy atoms). The largest differences were observed in the dynamically mobile parts of the structure: the elongated Cys24–Cys25 loop and the *C*-terminal helix (Asn41–Lys51) (Figure S9).

The resulting Phα1β structure is shown in Figure 4B. In addition to the system of six disulfide bonds, it is stabilized by 16 backbone–backbone hydrogen bonds. The ICK motif in the Phα1β molecule involves the antiparallel β-sheet formed by two strands (Asn20–Cys24 and Cys35–His39), connected by the elongated loop stabilized by the Cys24–Cys35 disulfide and with the turn of the 3_10_ helix (Ser29–Gly31) at its tip. The additional short β-strand is formed by the residues Glu7–Ile8 (Figure 4B). The *C*-terminal α-helix (Asn41–Cys52) is stapled to the ICK domain by the Cys18–Cys45, and Cys13–Cys52 disulfides, as well as by the Asp11–Lys51 and Asp12–Arg47 ionic bridges, the existence of which was suggested from the spatial proximity of the corresponding charged groups in the calculated set of structures (Figure 4C).

Comparison of the disulfide patterns revealed a close correspondence between the Phα1β and other spider toxins targeting the Ca^2+^ channels containing three and four disulfide bonds (Figure 1B,C). In addition, the disulfide pattern of Phα1β corresponds to the pattern of Spiderine-1a, the only spider knottin with a known spatial structure containing five disulfide bridges [33] (Figure 1A). An additional sixth disulfide, Cys18–Cys45, is one of the disulfides connecting the peptide *C*-terminus with the ICK motif. The Phα1β structure presented here is the first structure of the spider knottin, with six disulfide bridges per one ICK domain. Notably, there are double-knot toxins having six disulfides per two ICK domains, such as the specific TRPV1 agonist, DkTx [43], and the acid-sensing ion channel 1a (ASIC1a) inhibitor Hi1a [44]. The Phα1β structure represents a good reference for other toxins of the ctenitoxin family.

### 2.3. Backbone Dynamics of Phα1β 

To investigate the conformational plasticity of the Phα1β molecule, we measured the relaxation parameters of backbone the ^15^N nuclei (R_1_ and R_2_ relaxation rates and ^15^N–{^1^H} heteronuclear NOEs) at 60 MHz, pH 5.3, and 30 °C (Figure S10). To characterize the backbone motions on the ps–ns and μs–ms time-scales and describe the overall reorientation of the molecule in solution (ns time-scale), the relaxation data were analyzed using the so-called “model-free” approach. Calculations with an isotropic rotational diffusion tensor model gave an overall rotational correlation time (τ_R_) of ~3.2 ns. This value corresponds to the reorientation of a globular particle having a hydrodynamic Stokes radius (R_H_) ~16 Å, and is in good agreement with the dimensions of the Phα1β molecule (34 × 27 × 23 Å). Thus, the Phα1β molecule in solution is in the monomeric form.

**Figure 5:**
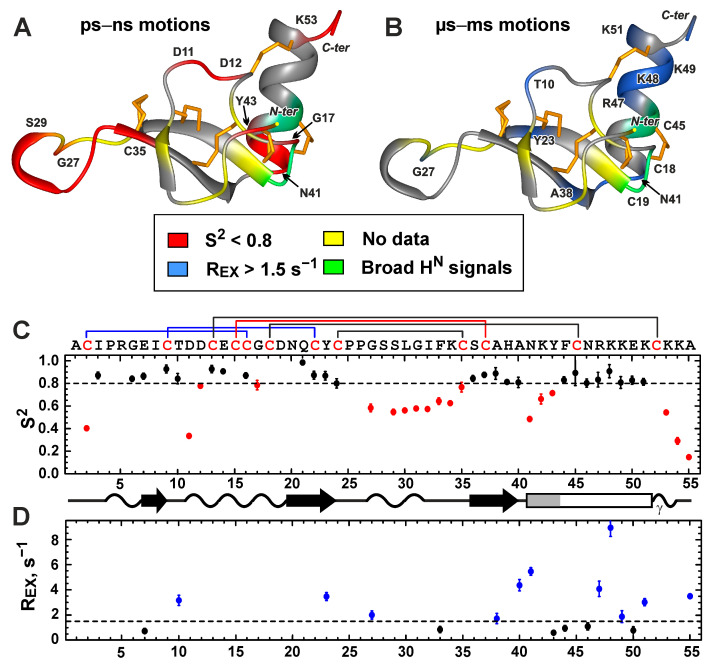
Backbone dynamics of Phα1β. (**A**,**B**) Regions with high-amplitude ps–ns and μs–ms mobility (S^2^ < 0.8 and R_EX_ > 1.5 s^−1^) are shown in red and blue, respectively. Sites where significant broadening of the ^1^H_N_ signals was observed are colored in green. Residues for which no dynamic data are available (*N*-terminal Ala residue, Pro residues, and spectral overlap) are highlighted in yellow. (**C**,**D**) The values of generalized-order parameters (S^2^) and exchange contributions to R_2_ relaxation rates (R_EX_), calculated during the “model-free” analysis of ^15^N relaxation data (60 MHz, pH 5.3, 30 °C). The data points with S^2^ < 0.8 and R_EX_ > 1.5 s^−1^ are colored in red and blue, respectively.

The low (<0.8) values of the squared generalized-order parameters (S^2^) revealed the regions of the toxin with a high mobility on the ps–ns time-scale (Figure 5A,C, red). High-amplitude motions were observed for the *N-* and *C*-terminal residues, the Asp11–Asp12 dipeptide fragment, the Cys24–Cys35 protruding loop, and on the first turn of the α-helix (Asn41–Tyr43). In the latter case, the motions were probably associated with fluctuation of the first helical turn between the α and 3_10_ conformation.

The presence of large (>1.5 s^−1^) exchange contributions to the R_2_ relaxation rates (R_EX_, Figure 5B,D, blue) and significant broadening of the HN signals (Figure 5B, green) revealed the regions of the molecule with high μs–ms mobility. High-amplitude motions at this time-scale were observed for the individual residues (Thr10, Tyr23, Gly27) near the sites of significant ps–ns motions (Asp11–Asp12 dipeptide and Cys24–Cys35 loop). We hypothesized that the flexibility of the backbone in the ps–ns time-scale contributes to the slower conformational fluctuations (switching between multiple conformations) of these residues. Other sites of extensive μs–ms fluctuations corresponded to the regions where the *C*-terminal helix joins the ICK domain. These sites included the loop between the third β-strand and α-helix (Ala38–Asn41), residues forming the Cys18–Cys45 disulfide, and the region of positively charged residues at the *C*-terminus of the α-helix (Arg47–Lys51), where two ionic bridges with the ICK domain were formed (Figure 5B). The observed pattern of the μs–ms fluctuations corresponds to the movement of the entire α-helix, relative to the rest of the peptide. The possible source of these fluctuations is an unusually high charge density at the *C*-terminus of the α-helix and in the nearby regions. Along with the Asp11–Lys51 and Asp12–Arg47 residues forming the ionic bridges, these regions also contain the Glu14, Glu50, and Lys48 residues (Figure 4C). The switching of the residues participating in the formation of the ionic bridges likely leads to the observed fluctuations in the position of the α-helix.

### 2.4. Properties of the Phα1β Surface 

**Figure 6:**
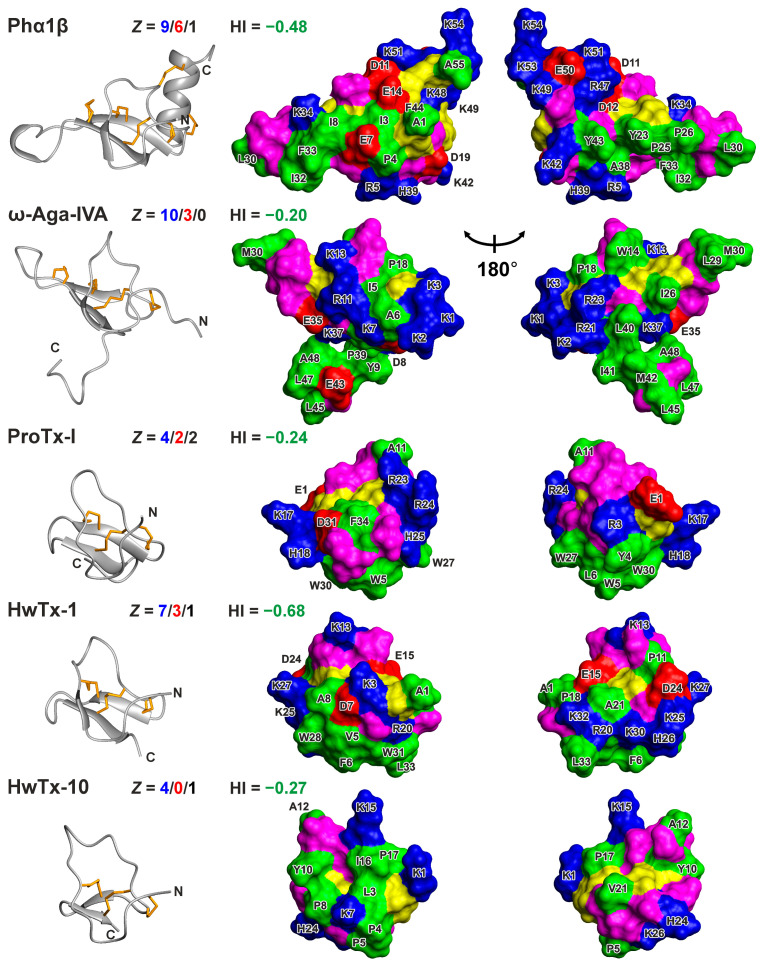
Comparison of Phα1β spatial structure with other inhibitors of Ca^2+^ channels from spider venom. Ribbon representation and two-sided view of Phα1β, ω-Agatoxin-IVA, ProTx-I, Huwentoxin-1, and Huwentoxin-10 surfaces. PDB codes are 8BWB (this work), 2NDB [45], 2M9L [23], 1QK6 [46], and 1Y29 [47], respectively. The molecules are superimposed over a C^α^ atom of six conserved Cys residues, which form the Cys knot. Hydrophobic (Ala, Met, Ile, Leu, Val, Phe, Trp, Tyr, and Pro), polar (Asn, Gln, Gly, Ser, and Thr), positively charged (Arg, Lys, and His), negatively charged (Asp and Glu), and Cys residues are colored green, magenta, blue, red, and yellow, respectively. For each of the toxins, the number (Z) of positively charged, negatively charged, and His residues are shown by blue, red, and black, respectively. Hydrophobicity indexes (HI or grand average of hydropathicity, GRAVY) were calculated using Kyte–Doolittle scale [48] in the Expasy ProtParam tool (https://web.expasy.org/protparam/ (accessed on 17 October 2022)). The HI values are shown in green. The maximum and minimum possible HI values are +4.5 and −4.5 for the poly-Ile and poly-Arg sequences, respectively.

Analysis of the Phα1β structure showed that the positively charged, negatively charged, and hydrophobic residues are segregated on the surface of the molecule (Figure 6 and Supplementary Figure S11). Most of the negatively charged groups (Asp11, Asp12, and Glu14) form a cluster on the peptide surface. Positively charged residues are grouped in two regions. The first region on the *C*-terminal helix includes the Arg47, Lys48, Lys49, Lys51, Lys53, and Lys54 side chains and the nearby *N*-terminal NH_3_^+^ group (Ala1). The second region located in the ICK domain includes the side chains of Arg5, Lys42, and His39 (Figure 6, the histidine residue can have partial positive charge at moderately acidic to neutral pH). At the same time, the most hydrophobic residues (Tyr23, Pro25, Pro26, Leu30, Ile32, and Phe33) are localized in the elongated Cys24–Cys35 loop protruding from the ICK core. Together with the cysteines and the Ile3, Pro4, Ile8, Tyr43, and Phe44 side chains, these residues form a belt around the Phα1β molecule, separating the two positively charged clusters on its surface (Figure 6 and Supplementary Figure S11).

The comparison of the surface properties of Phα1β with those of other spider toxins that inhibit voltage-gated Ca^2+^ channels (Figure 6) revealed a similar predominance of positively charged residues, but very different patterns of hydrophobic regions. Like Phα1β, all these toxins have a net positive charge (from +4 to +7, assuming that all histidines are positively charged) and a very similar overall hydrophobicity (Kyte–Doolittle index from −0.20 to −0.68), indicating that the peptides are moderately polar (Figure 6). In the membrane-active “promiscuous” gating modifier toxin, ProTx-I, which probably attacks the S1–S4 domain from the membrane side [24,27], the hydrophobic residues are gathered into one large cluster, occupying one side of the toxin molecule. A similar hydrophobic pattern is observed on the surface of Huwentoxin-1, which is also active on Ca^2+^ and Na^+^ channels [49]. A large hydrophobic region, formed by the unstructured *C*-terminal fragment, is also present in ω-Agatoxin-IVA (ω-Aga-IVA), which modifies the gating of the P-type Ca^2+^ channels (Ca_V_2.1) by binding to the S1–S4 domain, and also exhibits a weak membrane affinity [45,50]. Interestingly, the negatively charged Glu43 group protrudes in the center of this region. In contrast, Huwentoxin-10, which selectively blocks the N-type Ca^2+^ channels (Ca_V_2.2) by physical pore occlusion [47], exhibits the hydrophobic cluster on the other side of the ICK motif, and this cluster contains the positively charged Lys7 residue in its center (Figure 6).

The pattern of the hydrophobic residues in Phα1β resembles that observed in ω-Aga-IVA (Figure 6), assuming that the elongated hydrophobic region of Phα1β with the protruding negatively charged Glu7 residue is equivalent to the *C*-terminal fragment in ω-Aga-IVA with the protruding Glu43 side chain. In this case, the large positively charged cluster at the *C*-terminus of Phα1β may be equivalent to the group of positively charged residues located at the *N*-terminus of ω-Aga-IVA. This correspondence suggests that Phα1β may also target the S1–S4 voltage-sensing domains in the Ca^2+^ channels.

### 2.5. Interaction of Phα1β with Lipid Bilayers

To study the ability of Phα1β to interact with lipid bilayers, small (100 nm in diameter) unilamellar vesicles (SUVs) containing zwitterionic lipids (POPC) or a mixture of zwitterionic and negatively charged lipids (POPC/POPG 3:1) were used. Since electrostatic interactions may be important for the peptide/membrane interaction, we performed the measurements at pH = 7.0 to ensure that all ionizable groups were in a physiologically relevant state. In this case, the line-broadening, induced by the exchange with a solvent, did not interfere with the NMR measurements, since we only measured the overall envelope of the HN–aromatic region in the 1D ^1^H spectra.

**Figure 7:**
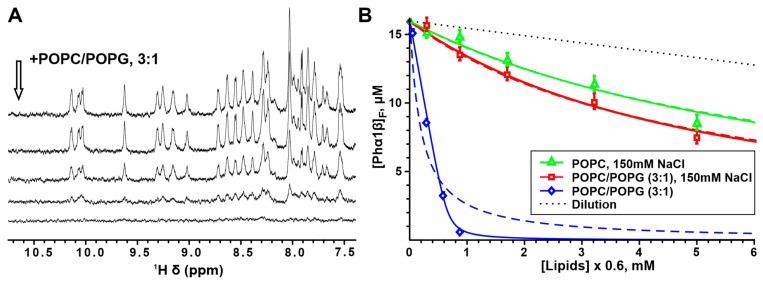
Binding of Phα1β to small unilamellar lipid vesicles (SUVs). (**A**) Amide–aromatic region of the 1D ^1^H NMR spectra of 16 μM Phα1β, measured upon the gradual addition of the POPC/POPG (3:1) vesicles (20 mM Tris–HCl, pH 7.0, 30 °C, 800 MHz). The arrow indicates the direction of increase in the lipid concentration. (**B**) Binding curves describing the interaction of Phα1β with POPC and POPC/POPG (3:1) SUVs are approximated by the partition equilibrium equation (dashed lines, Equation (1)) and the Langmuir isotherm (solid lines, Equation (2)). The fitted parameters are collected in Table 1.

The gradual addition of lipid vesicles to the Phα1β sample resulted in a gradual decrease in the intensity of the toxin signals in the 1D ^1^H NMR spectrum (Figure 7A). This effect was due to the peptide binding to the vesicle surface, but it can be explained by different mechanisms depending on the rate of exchange between the free and membrane-bound toxins. The toxin molecules bound to the lipid vesicles are unobservable through NMR spectroscopy due to the very slow reorientation of the vesicles in solution and the very large signal linewidths. With the limit of the fast or intermediate exchange (on the NMR time-scale), the toxin binding to the vesicles should lead to a significant broadening of the free toxin signals. At the same time, with the limit of a slow exchange, the signals of free toxin should not show broadening. Indeed, all spectra obtained in the presence of lipid vesicles were adequately approximated by the intensity-scaled spectrum of lipid-free Phα1β (Table S2, Figure S12). Thus, the membrane binding of Phα1β only reduced the intensity of the toxin resonances, without significant line-broadening, and a slow exchange took place. In this case, the observed signal intensity is proportional to the concentration of free toxin in the aqueous phase, and the concentration of membrane-bound toxin can be calculated.

The comparison of the measured binding curves showed that Phα1β had a weak affinity for zwitterionic and partially anionic lipid membranes at an ionic strength close to physiological values (150 mM NaCl, Figure 7B, green and red curves). At the same time, under conditions of low ionic strength, the peptide demonstrated a much higher affinity for the negatively charged (POPC/POPG) membrane (Figure 7B, blue curve). Therefore, the positively charged Phα1β molecule (total charge +4) binds to the lipid bilayer mainly through electrostatic interactions, while hydrophobic interactions play a minor role.

The measured binding isotherms were approximated by two models: the partition equilibrium and Langmuir surface adsorption. Both models described the data measured in 150 mM NaCl equally well, while at a low ionic strength, the two-parameter Langmuir model performed much better than the one-parameter partition equilibrium model (Figure 7B). Nevertheless, for comparison with the literature data, we presented the parameters obtained for both models in Table 1.

The *K_N_* parameter extracted by fitting the data with the Langmuir model indicates a relatively high affinity of Phα1β to the surface of the POPC/POPG vesicles in the absence of salt (Table 1). At the same time, a very large value of the parameter *N* (~40) revealed that this high-affinity interaction is the result of the summarized multiple weak interactions with individual lipid molecules.

The comparison of the obtained data with the results of the previous study of the membrane-active voltage-sensor spider toxin Hm-3 targeting Na^+^ channels [28] shows that Phα1β interacts with the lipid membranes under similar conditions, with a much lower affinity. For example, the partition coefficients, *K_p_,* of Hm-3 for the POPC/DOPG (3:1), POPC/DOPG (3:1, 150 mM NaCl), and POPC (150 mM NaCl) membranes were 12.5, 8.6, and 9.5 × 10^3^·M^−1^, respectively. These values indicate that, in contrast to Phα1β, some membrane-active voltage-sensor toxins do not completely lose their membrane activity under physiological ionic conditions, and show a moderate affinity for uncharged membranes.

The comparison with the data measured for a series of voltage-sensor spider toxins acting on the low-voltage-activated Ca^2+^ channels [51] and a series of spider toxins acting on K^+^ and Na^+^ voltage-gated channels (including ProTx-I) [26,27] also confirms the above conclusion. In these works, the partition coefficients have been measured in the slightly different lipid system: large unilamellar vesicles (LUVs) composed of POPC/POPG (1:1) at a low ionic strength. The reported *K_p_* values were significantly higher than those for Phα1β (181, 110, 137, 18, 20, 199, 87, and 62 × 10^3^·M^−1^ for ProTx-II, PaTx-1, GsAF-I, GsAF-II, GxTx-1E, ProTx-I, Hanatoxin, and SGTx1 toxins, respectively). Note that the literature *K_p_* values were recalculated according to the definition of the partition coefficient used in this work (see Section 4). Interestingly, the *K_p_* values for Hanatoxin and SGTx1 were reduced to 9.6 and 2.0 × 10^3^·M^−1^, respectively, with the addition of 100 mM K+ ions. This reduction was similar to that observed for Phα1β upon increasing ionic strength (Table 1).

The observed weak binding of Phα1β to zwitterionic lipid membranes does not contradict the possible toxin interaction with the S1–S4 voltage-sensing domains. Indeed, the classic membrane-active gating-modifier toxin, VsTx1 (overall charge +3), also does not show an affinity for uncharged membranes in vitro [52]. However, taking into account that the outer leaflet of neuronal membranes contains a detectable fraction (~3%) of anionic lipids represented by glycolipids [53]. Thus, toxins such as Phα1β or VsTx1 can interact with neuronal membranes in vivo. The most similar partition coefficient values to those of Phα1β were previously observed for the ω-Aga-IVA toxin (14.2 and 0.1 × 10^3^·M^−1^ in the POPC/POPG (1:1) and POPC liposomes, respectively) [45]. This observation supports the above suggestion that Phα1β may act as a gating modifier of the Ca^2+^ channels.

### 2.6. Interaction of Phα1β with Rat TRPA1 Expressed in Xenopus Oocytes

**Figure 8:**
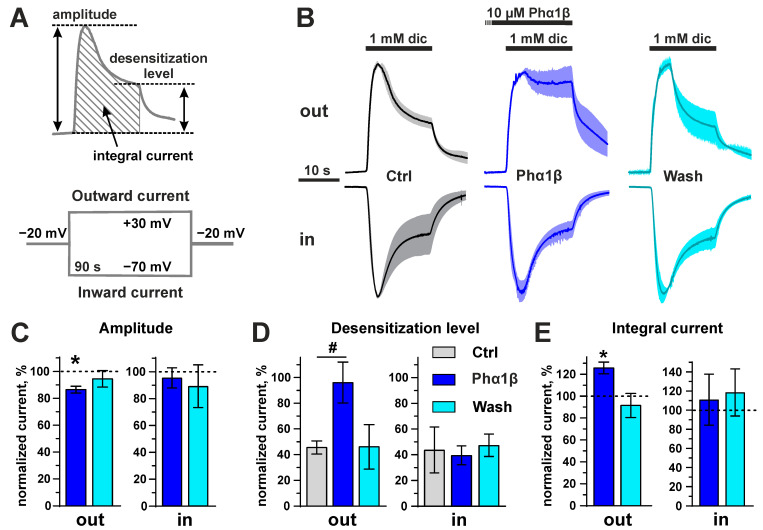
Recombinant Phα1β affects the outward diclofenac-evoked TRPA1 currents in *X. laevis* oocytes in the experiments with rectangular voltage pulses. (**A**) Scheme of the experiments and properties of the current traces quantitatively analyzed in the panels (**C**–**E**). (**B**) Average current traces normalized by the amplitude of the control (“Ctrl”) current of the same direction (n = 3, different oocytes were recorded, S.E.M. range is shown as the shade around the trace). The application of compounds is shown by bars above the current traces (30 s pre-incubation phase for Phα1β is shown off-scale). Diclofenac (1 mM) and Phα1β (10 µM) were used. The direction of the current is shown by the “Out” and “In” labels. (**C**) The mean current amplitude normalized to the amplitude of the control currents (100%). * *p* < 0.05 indicates a significant difference between the “Phα1β” data group and control (100%) using a one-sample two-sided t-test. (**D**) The mean level of the desensitized current normalized to the current amplitude in each trace (100%). # *p* < 0.05 indicates a significant difference between the “Phα1β” and “Ctrl” data groups using a two-sided t-test. Data in the “Wash” group were not statistically analyzed. (**E**) The mean integral current normalized to the integral currents in the “Ctrl” traces (100%). * *p* < 0.05 indicates a significant difference between the “Phα1β” data group and control (100%) using a one-sample two-sided t-test. Data are shown as the mean ± S.E.M. (*n* = 3, recorded in different oocytes).

**Figure 9:**
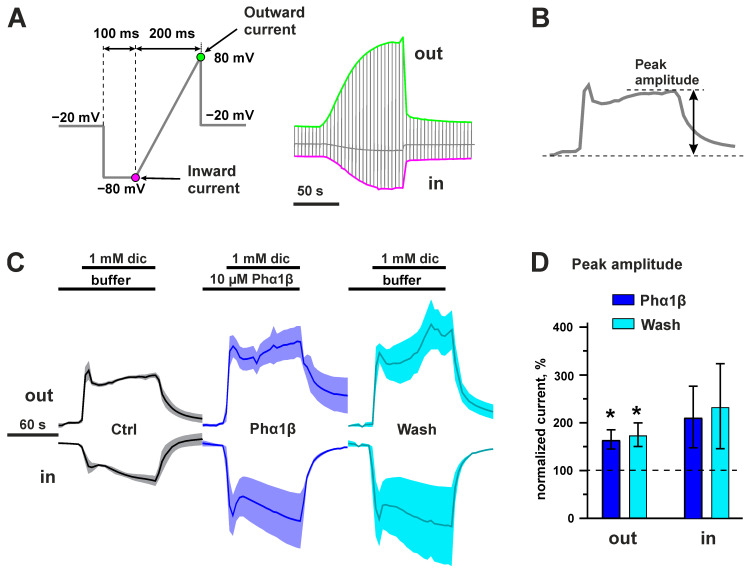
Recombinant Phα1β affects diclofenac-evoked TRPA1 currents in *X. laevis* oocytes in the experiments with voltage ramps. (**A**) Scheme of the experiments and parameters of the membrane voltage ramp used for the inward and outward current recordings. The moments of outward and inward current recordings are shown by color dots. The example of the resulting trace is shown on the right. (**B**) Peak amplitude of current trace quantitatively analyzed in the panel (**D**). (**C**) Average current traces normalized by the peak amplitude of the control current (‘Ctrl’) of same direction (n = 3–5, different oocytes were recorded, S.E.M. range is shown as the shade around the trace). The application of compounds is shown by bars above the current traces. Diclofenac (1 mM) and Phα1β (10 µM) were used. The direction of the current is shown by the “Out” and “In” labels. (**D**) The mean peak amplitudes normalized to the amplitude of the “Ctrl” currents (100%). * *p* < 0.05 indicates a significant difference between the data groups and control (100%) using a one-sample two-sided t-test. Data are shown as the mean ± S.E.M. (*n* = 3–5, recorded in different oocytes).

To characterize the interaction of Phα1β with TRPA1, we studied the effect of the recombinant toxin on *Xenopus laevis* oocytes expressing the rat channel. We used two different approaches for electrophysiological recordings: (1) the rectangular pulses of voltage from the holding potential (−20 mV) to +30 or −70 mV for recording of outwards or inward currents, respectively (Figure 8A), and (2) fast ramps, from −80 mV to +80 mV, for recording the currents in both directions in one experiment (Figure 9A). The second approach is frequently used for TRPA1 studies in *Xenopus* oocytes (see [54,55]). In both cases, the application of Phα1β itself did not produce any currents and changes in the leakage currents.

In the experiments of first type, the 30 s preincubation with 10 µM Phα1β resulted in the moderate (to 86 ± 3%) decrease in the amplitude of the outward currents evoked by non-covalent agonist (diclofenac). This effect was reversible (Figure 8B,C). The desensitization of the ion channel under the prolonged (30 s) agonist application was significantly affected by the co-application of 10 µM Phα1β: the current amplitude at the end of the agonist application significantly increased from 46 ± 5% (control) to 96 ± 16% (Phα1β, Figure 8B,D). This effect was also reversible. Therefore, the toxin moderately inhibited the amplitude of the outward ion currents through the TRPA1 channel, and simultaneously blocked its desensitization. These two effects oppositely influence the integral ion currents through the channel. As a result, 10 µM Phα1β significantly increased (to 126 ± 5%) the integral outward ion currents through the TRPA1 channel during the 30 s diclofenac application (Figure 8E). In the experiment with rectangular voltage pulses, Phα1β did not affect the parameters of diclofenac-evoked inward ion currents (Figure 8).

**Figure 10:**
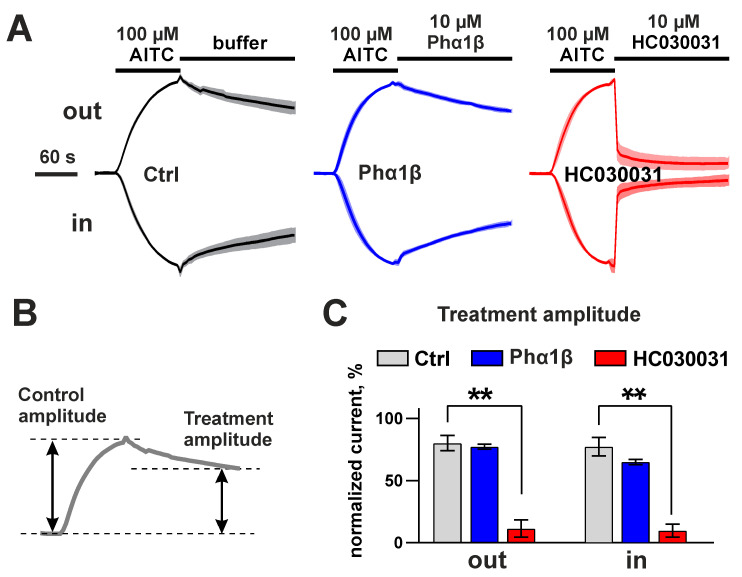
Recombinant Phα1β does not affect AITC-evoked TRPA1 currents in *X. laevis* oocytes in the experiments with voltage ramps. (**A**) Average current traces normalized by the amplitude of the control current, “Ctrl”, of the same direction (n = 5, different oocytes were recorded, S.E.M. range is shown as the shade around the trace). The application of the compounds is shown by the bars above the current traces. AITC (100 µM), Phα1β (10 µM), and HC030031 (50 µM) were used. The direction of the current is shown by the “Out” and “In” labels. (**B**) Definition of “treatment amplitude” is quantitatively analyzed in panel (**C**). (**C**) The mean “treatment amplitudes” normalized to the control amplitude in the same trace (100%). ** *p* < 0.01 indicates a significant difference between the “Ctrl” and “HC030031” data groups using a two-sided t-test.

In the experiment with the voltage ramps, the 30 s preincubation with 10 µM Phα1β increased the peak amplitude of the outward and inward diclofenac-evoked currents to 150–200%, and this effect was irreversible (Figure 9C,D). The stimulation with the covalent TRPA1 agonist, AITC, also produced measurable currents (Figure 10). These currents were not affected by the application of recombinant Phα1β after AITC stimulation, providing responses similar to the control (application of the ND-96 buffer). At the same time, the AITC-induced currents were completely inhibited by the TRPA1-specific inhibitor, HC030031 (Figure 10A,C).

The observed potentiation of the diclofenac-evoked ion currents and lack of the effects on the AITC-evoked currents is inconsistent with the previously published results of the Phα1β study on human TRPA1 expressed in HEK293 cells, or natively presented in IMR90 fibroblasts and DRG neurons [15]. In that work, the complete inhibition of the AITC-evoked inward Ca^2+^ currents by 10 µM Phα1β was observed in the calcium-imaging assay. To check the possible role of interspecies differences in the TRPA1 structure (rat vs. human), we tested our recombinant Phα1β on human TRPA1 expressed in *Xenopus* oocytes. As with the rat channel, no effects were observed on AITC-evoked outward and inward currents (data not shown). We hypothesize that the reason for the observed discrepancy can be a prolonged (10 m) preincubation of the cells with the toxin used in the previous work [15]. Phα1β likely potentiates the TRPA1 channel, as seen in our experiments with diclofenac, but the potentiation leads to the channel inactivation (desensitization), manifested as inhibition, during a long preincubation period. The lack of effects on the AITC-evoked currents (Figure 10) suggests that Phα1β cannot potentiate the channels already activated by the covalent agonist.

Unfortunately, we were unable to adapt the published protocol [15] to recordings in *Xenopus* oocytes using both the toxin preincubation and AITC stimulation. The difference in the TRPA1 expression renders the AITC responses in *Xenopus* oocytes heterogeneous and requires a separate normalization of the data on the amplitude of the control response. On the other hand, the covalent attachment of AITC to the channel prevents the dissociation of the agonist and makes it impossible to register the control and test responses from the same cell. There is only one possibility: to register the control and test responses within the same agonist stimulus, implying the initial application of AITC and the subsequent application of the test compound (Figure 10).

The observed TRPA1 potentiation does not contradict the analgesic properties of Phα1β. Compounds that potentiate the TRPA1 channel may also demonstrate analgesic properties [54,55]. It is likely that Phα1β induces the desensitization of the neurons expressing TRPA1, which in turn causes a significant decrease in nociceptive and inflammatory responses.

The outer side of the TRPA1 channel contains only a few sites where toxins can potentially bind. The modulation of the channel desensitization (Figure 8) and potentiation of the agonist response (Figure 9) imply that Phα1β does not physically occlude the ion-conducting pore of TRPA1. Thus, we can consider Phα1β as a gating modifier toxin that likely interacts with the outer interface of the S1–S4 domain. This suggestion is consistent with other properties of Phα1β that are similar to those of the gating modifiers, such as “promiscuous” targeting of unrelated ion channels (Ca_V_2 and TRPA1) and membrane-binding.

## 3. Conclusions

In contrast to the previous data [34], the NMR study showed that the Phα1β molecule is stabilized by six disulfide bonds with the arrangement of Cys1–5, Cys2–7, Cys3–12, Cys4–10, Cys6–11, and Cys8–9. The Phα1β molecule consists of two domains: a *N*-terminal inhibitory cystine knot (ICK) and a *C*-terminal helical domain, connected by two disulfides and a system of salt bridges. The ICK domain, stabilized by four disulfide bonds, contains a long protruding loop. The ^15^N relaxation data and the absence of the interdomain NOE contacts revealed a rather weak connection between the domains, which fluctuate relative to each other, with a characteristic time in the micro–millisecond range. The clustering of the charged and hydrophobic residues on the Phα1β surface and the toxin affinity for partially anionic lipid vesicles were described. The Phα1β structure is the first determined structure of spider knottin, with six disulfide bridges per one ICK domain.

In addition, electrophysiological recordings in *Xenopus* oocytes revealed a significant potentiation and blocking of the desensitization of the TRPA1 channel by Phα1β, contrary to previous data [15]. This modulating behavior is consistent with the toxin binding to some regulatory site on the channel molecule, but not in the pore vestibule. Summarizing our findings, we suggest that Phα1β is a typical gating modifier toxin, characterized by the membrane affinity and targeting S1–S4 gating domains in unrelated ion channels (Ca_V_2 and TRPA1).

## 4. Materials and Methods

### 4.1. Bacterial Expression of Phα1β

The gene encoding 55 amino acid residues of the Phα1β toxin from *P. nigriventer,* with an additional ATG codon at the 5′-end, was constructed from overlapping synthetic oligonucleotides using PCR and considering the codon frequency in *E. coli*. The *Phα1β* gene was cloned into the *pET-32a(+)* expression vector (Novagen) at the *Nco*I and *Hin*dIII restriction sites. SHuffle *E. coli* cells, transformed by the *pET-32a(+)/Phα1β* vector, were grown at 37 °C on a TB medium (12 g of bacto-tryptone, 24 g of yeast extract, 4 mL of glycerol, 2.3 g of KH_2_PO_4_, 12.5 g of K_2_HPO_4_ per 1L of medium, pH 7.4) in the presence of ampicillin (100 μg/mL). The expression of the TRX–Phα1β fusion protein gene was induced by adding isopropyl β-D-1-thiogalactopyranoside (IPTG) to the final concentration of 0.1 mM at the OD_600_ of 0.6. After the induction, the cells were grown at 13 °C for 72 h. In order to produce ^15^N-labeled Phα1β, the transformed SHuffle cells were cultured on an LB medium to a cell density OD_600_ of 0.6. The biomass was centrifuged at 3000× *g* for 20 min. The cell pellet was aseptically resuspended in a three-times smaller volume of M9 minimal medium (6 g of Na_2_HPO_4_, 3 g of KH_2_PO_4_, 0.5 g of NaCl, 2 g of ^15^N NH_4_Cl (Cambridge Isotope Laboratories, Tewksbury, MA, USA), 240 mg of anhydrous MgSO_4_, 11 mg of CaCl_2_, 2 g of Glycerol, 2 mg of yeast extract, and 200 μL of 5% thiamine chloride per 1 L of medium, pH 7.4, 100 μg/mL ampicillin) to a final OD_600_ of 1.8, and the gene expression was induced by the addition of 0.1 mM IPTG. Further cultivation conditions were similar to those described on the TB medium.

The cells from 1 L of culture were harvested via centrifugation (10,000× *g*, 20 min, 4 °C) and resuspended in 50 mL of buffer A (30 mM Tris–HCl, 0.3 M NaCl, pH 8.0), supplemented with 0.5 mg/mL lysozyme and 1 mM PMSF. The cell suspension was disintegrated by ultrasound for 6 min and centrifuged at 30,000× *g* for 30 min. The cell lysate was loaded on the 4 mL Ni–Sepharose 6 Fast-Flow column, preliminarily equilibrated with the buffer A. The TRX–Phα1β fusion protein was eluted using 250 mM imidazole. The samples containing TRX–Phα1β were acidified by the addition of up to 0.3 M HCl, as described in [56]. The cleavage of the TRX–Phα1β fusion construct was achieved through the addition of 100 molar excess of BrCN, followed by incubation at room temperature in the dark for 16–24 h. The samples were dried under vacuum and purified by reverse-phase HPLC. Chromatography was carried out on Jupiter C4, column (A300, 4.6 × 250 mm, Phenomenex). Phα1β was eluted with the acetonitrile gradient 90–45%, in the presence of 0.1% TFA for 50 min; the resulting Phα1β sample was lyophilized.

### 4.2. Liquid Chromatography and Mass Spectrometry

The sample was loaded on a home-made trap column 50 × 0.1 mm, packed with Inertsil ODS3 3 μm (GL Sciences, Tokyo, Japan) sorbent (Dr. Maisch, Ammerbuch-Entringen, Germany), in the loading buffer (2% acetonitrile, 98% H_2_O, 0.1% TFA) at a 4 μL/min flow, and was separated at an RT in a home-packed [57] fused-silica column 300 × 0.1 mm packed with Reprosil PUR C18AQ 1.9 (Dr. Maisch) into an emitter prepared with P2000 Laser Puller (Sutter, Novato, CA, USA). Reverse-phase chromatography was performed with an Ultimate 3000 Nano LC System (Thermo Fisher Scientific), which was coupled to the Q Exactive Plus Orbitrap mass spectrometer (Thermo Fisher Scientific) via a nanoelectrospray source (Thermo Fisher Scientific). The peptides were loaded in a loading solution (98% 0.1% (*v*/*v*) formic acid, 2% (*v*/*v*) acetonitrile) and eluted with a linear gradient: 10% B for 3 min; 10–60% B for 12 min, 60–70% B for 4 min, 70–80% B for 1 min, 80% B during 3 min, 80–10% B for 0.1 min, at a flow rate of 500 nL/min. Buffer A comprised 5% acetonitrile and 0.1% formic acid, and buffer B 80% acetonitrile and 0.1% formic acid. The MS1 parameters were as follows: 140 K resolution, 500–2000 scan range, max injection time—200 msec, AGC target—3 × 10^6^.

### 4.3. NMR Experiments and Spatial Structure Calculation

The NMR experiments were performed using the samples containing 1.7 mM non-labeled or 0.27 mM ^15^N-labeled Phα1β in 5% D_2_O at a pH of 4.5. The NMR spectra were acquired on a Bruker Avance-III 600 spectrometer equipped with a cryoprobe, and Bruker Avance 700 spectrometer equipped with room-temperature probe at 30 °C. The resonance assignment was performed using a standard approach based on 2D ^1^H–^15^N-HSQC, ^15^N-filtered 3D TOCSY-HSQC (τ_m_ = 80 μs), and 3D NOESY-HSQC (τ_m_ = 100 μs) spectra measured for the ^15^N-labeled peptide, and 2D NOESY (τ_m_ = 100 μs) and 2D TOCSY (τ_m_ = 60 μs) spectra measured for the unlabeled sample. The partial ^13^C assignment was performed using a 2D ^1^H-^13^C-HSQC spectrum measured at a natural isotope abundance. The ^3^J_H_^N^_H_^α^ and ^3^J_H_^β^_N_ scalar coupling constants were measured using 3D HNHA and HNHB spectra [58]. Additionally, ^3^J_H_^N^_H_^α^ and ^3^J_H_^β^_H_^α^ scalar couplings were estimated via line-shape analysis in a 2D TOCSY spectrum. The 3D TOCSY-HSQC, HNHA, and HNHB spectra were acquired using a non-uniform sampling method, with 30% sparse sampling and processed with MDDNMR [59]. The temperature gradients of the amide protons (∆δ ^1^H^N^/∆T) were measured from a series of ^15^N-HSQC spectra acquired in the 15–45 °C temperature range.

The secondary structure of Phα1β was calculated from ^1^H, ^13^C, and ^15^N chemical shifts using TALOS-N [39]. For the 3D structure calculation, the distance constraints were derived from cross-peak intensities in 2D/3D NOESY spectra (τ_m_ = 100 ms). The φ and χ1 dihedral angle restraints were obtained from J-couplings and NOE intensities. The hydrogen bonding restraints were applied, assuming that an amide proton with ∆δ ^1^H^N^/∆T > −4.5 ppb/K can participate in the hydrogen bonding. The standard distance restraints (implemented in CYANA) were applied to restrain the disulfide connectivity. The 3D structures were calculated using CYANA ver. 3.98 [41]. The visualization and analysis of the calculated structures were performed using MOLMOL [60].

The relaxation parameters of the ^15^N nuclei (longitudinal (R1), transverse (R2) relaxation rates, and steady-state heteronuclear ^15^N–{^1^H} NOEs) were measured at 60 MHz, a pH of 5.3, and 30 °C, using the standard set of ^15^N-HSQC-based pseudo-3D experiments. The relaxation data were analyzed using FastModelFree [61]. An isotropic diffusion model was used.

### 4.4. Liposome Titration

The lipid stock solutions for the preparation of liposomes were prepared by hydrating dry lipids, POPC and POPG (Avanty Polar Lipids, Birmingham, AL, USA), using 20 mM Tris buffer at a pH of 7.0, with or without 150 mM NaCl. Small unilamellar vesicles were prepared by extrusion through a 100 nm PTFE membrane (Whatman, Cleves, OH, USA). The lipid concentrations were confirmed using 1D ^1^H NMR by dissolving a small amount of the lipid solution in the CDCl_3_/CD_3_OD/D_2_O (16:7:1) mixture. An SUV suspension (50 mM) was gradually added to the 16 μM Phα1β sample in the same buffer. At each lipid concentration, a 1D ^1^H NMR spectrum was acquired at 30 °C. The toxin concentration in the aqueous phase (non-bound to liposomes) was measured by fitting the Phα1β spectrum without lipids to the spectra acquired in the presence of lipids. The 1D ^1^H NMR spectra were processed with 32,768 complex points for a full spectral width (20 ppm), and the amide–aromatic regions (7.5–11.0 ppm) were extracted for analysis. In each case, the parameters *k* and *b* were optimized using the least-squares method to find the best approximation of the expression ${k\cdot \overset{-}{S}}_{0}+b={\overset{-}{S}}_{L}$, were ${\overset{-}{S}}_{0}$ and ${\overset{-}{S}}_{L}$, i.e., the vectors containing intensities from the Phα1β spectra measured in the absence and presence of lipids, respectively. The obtained values of parameter *k* were recalculated according to the concentration of the free toxin in the solution, using a known total concentration of the toxin in the sample.

To quantify Phα1β binding, the data were approximated by two models: partition equilibrium between the aqueous and lipid phases, as shown below,

(1) $$K_{p}\cdot C_{f}=C_{b}/L^{\prime }$$

and Langmuir surface adsorption

(2) $$\frac{1}{K_{N}}=\frac{C_{f}\cdot \left(L\prime -N\cdot C_{b}\right)}{N\cdot C_{b}}$$

were *C_f_* and *C_b_* are the toxin concentrations in the aqueous and lipid phases, respectively, *K_P_* is the partition coefficient, *K_N_* is the affinity constant of the peptide to the site on the vesicle surface formed by *N* lipid molecules, and *L′* is the lipid concentration in the outer leaflet of the vesicles (60% of total lipid; *L′* = 0.6 × *L* [62]). During the analysis, the effect of the dilution was taken into account. Calculations were performed using the Mathematica 12.2 software (Wolfram research, Champaign, IL, USA).

### 4.5. Electrophysiological Recordings

The rat TRPA1 receptor [55] was expressed in *X. laevis* oocytes, according to standard procedure. The preparation of the *Xenopus* oocytes at defolliculated stages V–VI was carried out as previously described [63]. The mRNA transcript encoding TRPA1 was synthesized by the mMessage mMachine T7 kit (Cat# AM1344, Thermo Fisher Scientific), according to the protocol for capped transcripts supplied by the manufacturer. Defolliculated oocytes were injected with 20 ng of mRNA and kept for 3–7 days at 18 °C in modified Barth’s solution, supplemented with gentamycin (Cat# G1264, Merck, Germany) (50 μg/mL) and containing (in mM) 88 NaCl, 1 KCl, 2.4 NaHCO_3_, 0.82 MgSO_4_, 0.33 Ca(NO_3_)_2_, 0.41 CaCl, and 5 HEPES, at a pH of 7.4.

Two-electrode voltage-clamp recordings were performed using the TEC-03X amplifier (NPI Electronics GmbH, Tamm, Germany). Prolonged clamping at the positive potentials (Vm) was found to increase the oocyte leakage current and decrease viability, so the holding and inter-pulse (sweep) Vm was set close to the mean oocyte resting potential (−20 mV). For rectangular voltage pulses, the Vm magnitudes during the pulse recording were set so they produced currents of similar amplitudes for both the outward and inward current directions, at +30 and −70 mV, respectively. For experiments with voltage ramps, the recording of inward/outward currents were made at repeated steps to −80 mV for 100 ms, following the voltage ramp from −80 mV to +80 mV for 200 ms every 4 s. The current amplitudes were measured at the beginning and the end of each voltage ramp [55].

Glass microelectrodes were pulled to ~1 MOhm resistance and filled with 3M KCl. The output currents and voltage signal were filtered at 50 Hz and digitized at 1 kHz by National Instruments USB-6251 card. The voltage ramps were triggered by an analog output from the same card and the ramp signals were generated by the KMoon FY6900 waveform generator connected to the command input of the amplifier. The data recording and perfusion system control were carried out via WinWCP 5.2.7 (Strathclyde Electrophysiology Software, Glasgow, UK). During the recoding, the oocytes were perfused at 2 mL/min with an ND-96 solution w/o Ca^2+^, containing (in mM) 96 NaCl, 2 KCl, 1 MgCl_2_, and 10 HEPES, at a pH of 7.4. The recordings were performed at room temperature (21–22 °C).

The currents were elicited by an exchange of the solution in the recording bath to an ND-96 solution with 1 mM diclofenac (Hemofarm a.d., Vršac, Serbia) [64] or 100 µM AITC (Sigma-Aldrich, Darmstadt, Germany) [65], and repeated with 5 min intervals. If necessary, the oocytes were pre-incubated for 30s in a 10 µM solution of recombinant Phα1β, and the current was stimulated by the Phα1β+diclofenac solution. In some experiments, the solution in the recording bath was exchanged to an ND-96 solution with 10 µM of recombinant Phα1β or 10 µM HC030031 (Sigma). The Phα1β and diclofenac solutions were prepared before each oocyte recording from a 300 µM stock of Phα1β and 84.4 mM stock of diclofenac by diluting with Ca^2+^-free ND-96.

The recorded data were processed in Clampfit 10.7 (Molecular Devices, San Jose, CA, USA) and analyzed in GraphPad Prism 8.0.1 (GraphPad Software, San Diego, CA, USA).

## Figures and Tables

**Table 1:** Energetic and stoichiometric parameters of Phα1β interaction with the SUVs obtained using the partition equilibrium equation (Equation (1)) and Langmuir isotherm (Equation (2)).

Lipids	Partition Equilibrium ^a^ *Kp*	Langmuir Isotherm ^b^	
		*K_N_*	*N*
	×10^3^·M^−1^	×10^6^·M^−1^	
POPC/POPG (3:1)	5.1 ± 0.1	2.8 ± 0.7	41 ± 1
POPC/POPG (3:1), 150 mM NaCl	0.20 ± 0.02	0.0087 ± 0.0046	41 ^c^
POPC, 150 mM NaCl	0.14 ± 0.01	0.0060 ± 0.0013	41 ^c^

^a^ *Kp* is the partition coefficient. The concentration of the “non-aqueous” phase was taken to be equal to the lipid concentration in the outer leaflet of the vesicles (60% of total lipid). ^b^ *K_N_* is the affinity constant of the peptide to the site on the vesicle surface formed by *N* lipid molecules. ^c^ This parameter was fixed in analogy with the POPC/POPG (3:1) system.

## Data Availability

Experimental restraints and atomic coordinates for the Phα1β have been deposited in PDB, under accession code 8BWB. The NMR chemical shifts were deposited in the BMRB database, under accession code 34776.

## Supplementary Materials

The following supporting information can be downloaded at: https://www.mdpi.com/article/10.3390/toxins15060378/s1, Figure S1: Full SDS-PAGE gel presented in Figure 2B. Figure S2: 1D ^1^H NMR spectra of unlabeled and ^15^N-labeled recombinant Phα1β. Figure S3: LC-MS analysis of the recombinant Phα1β. Figure S4: 2D TOCSY and NOESY NMR spectra of unlabeled recombinant Phα1β. Figure S5: 2D ^13^C-HSQC NMR spectrum of Phα1β. Figure S6: The fragment of 2D ^13^C-HSQC spectrum of Phα1β with resonances of prolines. Figure S7: The fragment of the 2D TOCSY spectrum of Phα1β with resonances of prolines. Figure S8: The fragment of the 2D NOESY spectrum of Phα1β with H^α^–H^δ^ cross-peaks for Xxx–Pro dipeptides. Figure S9: Comparison of an experimentally determined Phα1β structure with the model predicted by AlphaFold 2.0. Figure S10: ^15^N relaxation data for Phα1β, and the results of the “model-free” analysis [66]. Figure S11: Two-sided view of electrostatic and molecular hydrophobicity potentials on the Phα1β surface [67]. Figure S12: Fitting of the 1D ^1^H NMR spectra of the Phα1β measured in the presence of lipids. Table S1: Statistics for the best CYANA structures of Phα1β. Table S2: Residuals after the least-square fit of 1D ^1^H NMR spectra of the Phα1β measured in the presence of lipids.

## References

[1] Mouhat S., Jouirou B., Mosbah A., De Waard M., Sabatier J.-M. (2004). Diversity of Folds in Animal Toxins Acting on Ion Channels. Biochem. J..

[2] Herzig V., Cristofori-Armstrong B., Israel M.R., Nixon S.A., Vetter I., King G.F. (2020). Animal Toxins—Nature’s Evolutionary-Refined Toolkit for Basic Research and Drug Discovery. Biochem. Pharmacol..

[3] Pennington M.W., Beeton C., Galea C.A., Smith B.J., Chi V., Monaghan K.P., Garcia A., Rangaraju S., Giuffrida A., Plank D. (2009). Engineering a Stable and Selective Peptide Blocker of the Kv1.3 Channel in T Lymphocytes. Mol. Pharmacol..

[4] Morsy M.A., Gupta S., Dora C.P., Jhawat V., Dhanawat M., Mehta D., Gupta K., Nair A.B., El-Daly M. (2023). Venoms Classification and Therapeutic Uses: A Narrative Review. Eur. Rev. Med. Pharmacol. Sci..

[5] Lauria P.S.S., Villarreal C.F., Casais-e-Silva L.L. (2020). Pain Modulatory Properties of Phoneutria Nigriventer Crude Venom and Derived Peptides: A Double-Edged Sword. Toxicon.

[6] Maatuf Y., Geron M., Priel A. (2019). The Role of Toxins in the Pursuit for Novel Analgesics. Toxins.

[7] Cordeiro M.d.N., de Figueiredo S.G., Valentim A.d.C., Diniz C.R., von Eickstedt V.R., Gilroy J., Richardson M. (1993). Purification and Amino Acid Sequences of Six Tx3 Type Neurotoxins from the Venom of the Brazilian “armed” Spider Phoneutria Nigriventer (Keys). Toxicon.

[8] Gomez M.V., Kalapothakis E., Guatimosim C., Prado M.A.M. (2002). Phoneutria Nigriventer Venom: A Cocktail of Toxins That Affect Ion Channels. Cell Mol. Neurobiol..

[9] Souza A.H., Ferreira J., Cordeiro M.d.N., Vieira L.B., De Castro C.J., Trevisan G., Reis H., Souza I.A., Richardson M., Prado M.A.M. (2008). Analgesic Effect in Rodents of Native and Recombinant Ph Alpha 1beta Toxin, a High-Voltage-Activated Calcium Channel Blocker Isolated from Armed Spider Venom. Pain.

[10] de Souza A.H., Lima M.C., Drewes C.C., da Silva J.F., Torres K.C.L., Pereira E.M.R., de Castro Junior C.J., Vieira L.B., Cordeiro M.N., Richardson M. (2011). Antiallodynic Effect and Side Effects of Phα1β, a Neurotoxin from the Spider Phoneutria Nigriventer: Comparison with ω-Conotoxin MVIIA and Morphine. Toxicon.

[11] Rigo F.K., Trevisan G., Rosa F., Dalmolin G.D., Otuki M.F., Cueto A.P., de Castro Junior C.J., Romano-Silva M.A., Cordeiro M.d.N., Richardson M. (2013). Spider Peptide Phα1β Induces Analgesic Effect in a Model of Cancer Pain. Cancer Sci..

[12] do Silva J.F., Binda N.S., Pereira E.M.R., de Lavor M.S.L., Vieira L.B., de Souza A.H., Rigo F.K., Ferrer H.T., de Castro Júnior C.J., Ferreira J. (2021). Analgesic Effects of Phα1β Toxin: A Review of Mechanisms of Action Involving Pain Pathways. J. Venom. Anim. Toxins incl. Trop. Dis..

[13] Vieira L.B., Kushmerick C., Reis H.J., Diniz C.R., Cordeiro M.N., Prado M.A.M., Kalapothakis E., Romano-Silva M.A., Gomez M.V. (2003). PnTx3-6 a Spider Neurotoxin Inhibits K+-Evoked Increase in [Ca^2+^]i and Ca^2+^-Dependent Glutamate Release in Synaptosomes. Neurochem. Int..

[14] Vieira L.B., Kushmerick C., Hildebrand M.E., Garcia E., Stea A., Cordeiro M.N., Richardson M., Gomez M.V., Snutch T.P. (2005). Inhibition of High Voltage-Activated Calcium Channels by Spider Toxin PnTx3-6. J. Pharmacol. Exp. Ther..

[15] Tonello R., Fusi C., Materazzi S., Marone I.M., De Logu F., Benemei S., Gonçalves M.C., Coppi E., Castro-Junior C.J., Gomez M.V. (2017). The Peptide Phα1β, from Spider Venom, Acts as a TRPA1 Channel Antagonist with Antinociceptive Effects in Mice. Br. J. Pharmacol..

[16] Julius D. (2013). TRP Channels and Pain. Annu. Rev. Cell Dev. Biol..

[17] Logashina Y.A., Korolkova Y.V., Kozlov S.A., Andreev Y.A. (2019). TRPA1 Channel as a Regulator of Neurogenic Inflammation and Pain: Structure, Function, Role in Pathophysiology, and Therapeutic Potential of Ligands. Biochem. Mosc..

[18] Manolache A., Babes A., Madalina Babes R. (2021). Mini-Review: The Nociceptive Sensory Functions of the Polymodal Receptor Transient Receptor Potential Ankyrin Type 1 (TRPA1). Neurosci. Lett..

[19] Koivisto A.-P., Belvisi M.G., Gaudet R., Szallasi A. (2022). Advances in TRP Channel Drug Discovery: From Target Validation to Clinical Studies. Nat. Rev. Drug. Discov..

[20] Giorgi S., Nikolaeva-Koleva M., Alarcón-Alarcón D., Butrón L., González-Rodríguez S. (2019). Is TRPA1 Burning Down TRPV1 as Druggable Target for the Treatment of Chronic Pain?. Int. J. Mol. Sci..

[21] Souza Monteiro de Araujo D., Nassini R., Geppetti P., De Logu F. (2020). TRPA1 as a Therapeutic Target for Nociceptive Pain. Expert Opin. Ther. Targets.

[22] Rigo F.K., Dalmolin G.D., Trevisan G., Tonello R., Silva M.A., Rossato M.F., Klafke J.Z., Cordeiro M.d.N., Castro Junior C.J., Montijo D. (2013). Effect of ω-Conotoxin MVIIA and Phα1β on Paclitaxel-Induced Acute and Chronic Pain. Pharmacol. Biochem. Behav..

[23] Gui J., Liu B., Cao G., Lipchik A.M., Perez M., Dekan Z., Mobli M., Daly N.L., Alewood P.F., Parker L.L. (2014). A Tarantula-Venom Peptide Antagonizes the TRPA1 Nociceptor Ion Channel by Binding to the S1-S4 Gating Domain. Curr. Biol..

[24] Agwa A.J., Henriques S.T., Schroeder C.I. (2017). Gating Modifier Toxin Interactions with Ion Channels and Lipid Bilayers: Is the Trimolecular Complex Real?. Neuropharmacology.

[25] Männikkö R., Shenkarev Z.O., Thor M.G., Berkut A.A., Myshkin M.Y., Paramonov A.S., Kulbatskii D.S., Kuzmin D.A., Sampedro Castañeda M., King L. (2018). Spider Toxin Inhibits Gating Pore Currents Underlying Periodic Paralysis. Proc. Natl. Acad. Sci. USA.

[26] Milescu M., Vobecky J., Roh S.H., Kim S.H., Jung H.J., Kim J.I., Swartz K.J. (2007). Tarantula Toxins Interact with Voltage Sensors within Lipid Membranes. J. Gen. Physiol..

[27] Milescu M., Bosmans F., Lee S., Alabi A.A., Kim J.I., Swartz K.J. (2009). Interactions between Lipids and Voltage Sensor Paddles Detected with Tarantula Toxins. Nat. Struct. Mol. Biol..

[28] Berkut A.A., Peigneur S., Myshkin M.Y., Paramonov A.S., Lyukmanova E.N., Arseniev A.S., Grishin E.V., Tytgat J., Shenkarev Z.O., Vassilevski A.A. (2015). Structure of Membrane-Active Toxin from Crab Spider Heriaeus Melloteei Suggests Parallel Evolution of Sodium Channel Gating Modifiers in Araneomorphae and Mygalomorphae. J. Biol. Chem..

[29] Shenkarev Z.O., Lyukmanova E.N., Paramonov A.S., Panteleev P.V., Balandin S.V., Shulepko M.A., Mineev K.S., Ovchinnikova T.V., Kirpichnikov M.P., Arseniev A.S. (2014). Lipid–Protein Nanodiscs Offer New Perspectives for Structural and Functional Studies of Water-Soluble Membrane-Active Peptides. Acta Nat..

[30] Redaelli E., Cassulini R.R., Silva D.F., Clement H., Schiavon E., Zamudio F.Z., Odell G., Arcangeli A., Clare J.J., Alagón A. (2010). Target Promiscuity and Heterogeneous Effects of Tarantula Venom Peptides Affecting Na+ and K+ Ion Channels. J. Biol. Chem..

[31] Middleton R.E., Warren V.A., Kraus R.L., Hwang J.C., Liu C.J., Dai G., Brochu R.M., Kohler M.G., Gao Y.-D., Garsky V.M. (2002). Two Tarantula Peptides Inhibit Activation of Multiple Sodium Channels. Biochemistry.

[32] Kornilov P., Peretz A., Lee Y., Son K., Lee J.H., Refaeli B., Roz N., Rehavi M., Choi S., Attali B. (2014). Promiscuous Gating Modifiers Target the Voltage Sensor of K(v)7.2, TRPV1, and H(v)1 Cation Channels. FASEB J..

[33] Nadezhdin K.D., Romanovskaia D.D., Sachkova M.Y., Oparin P.B., Kovalchuk S.I., Grishin E.V., Arseniev A.S., Vassilevski A.A. (2017). Modular Toxin from the Lynx Spider Oxyopes Takobius: Structure of Spiderine Domains in Solution and Membrane-Mimicking Environment. Protein Sci..

[34] Wormwood K.L., Ngounou Wetie A.G., Gomez M.V., Ju Y., Kowalski P., Mihasan M., Darie C.C. (2018). Structural Characterization and Disulfide Assignment of Spider Peptide Phα1β by Mass Spectrometry. J. Am. Soc. Mass. Spectrom..

[35] Lyukmanova E.N., Shulepko M.A., Shenkarev Z.O., Dolgikh D.A., Kirpichnikov M.P. (2010). In Vitro Production of Three-Finger Neurotoxins from Snake Venoms, a Disulfide Rich Proteins. Problems and Their Solutions (Review). Russ. J. Bioorganic Chem..

[36] Krishnarjuna B., Sunanda P., Villegas-Moreno J., Csoti A., Morales R.A.V., Wai D.C.C., Panyi G., Prentis P., Norton R.S. (2021). A Disulfide-Stabilised Helical Hairpin Fold in Acrorhagin I: An Emerging Structural Motif in Peptide Toxins. J. Struct. Biol..

[37] Platzer G., Okon M., McIntosh L.P. (2014). PH-Dependent Random Coil (1)H, (13)C, and (15)N Chemical Shifts of the Ionizable Amino Acids: A Guide for Protein PK a Measurements. J. Biomol. NMR.

[38] Schubert M., Labudde D., Oschkinat H., Schmieder P. (2002). A Software Tool for the Prediction of Xaa-Pro Peptide Bond Conformations in Proteins Based on 13C Chemical Shift Statistics. J. Biomol. NMR.

[39] Shen Y., Bax A. (2013). Protein Backbone and Sidechain Torsion Angles Predicted from NMR Chemical Shifts Using Artificial Neural Networks. J. Biomol. NMR.

[40] Frishman D., Argos P. (1995). Knowledge-Based Protein Secondary Structure Assignment. Proteins.

[41] Schmidt E., Güntert P. (2015). Automated Structure Determination from NMR Spectra. Methods Mol. Biol..

[42] Jumper J., Evans R., Pritzel A., Green T., Figurnov M., Ronneberger O., Tunyasuvunakool K., Bates R., Žídek A., Potapenko A. (2021). Highly Accurate Protein Structure Prediction with AlphaFold. Nature.

[43] Bohlen C.J., Priel A., Zhou S., King D., Siemens J., Julius D. (2010). A Bivalent Tarantula Toxin Activates the Capsaicin Receptor, TRPV1, by Targeting the Outer Pore Domain. Cell.

[44] Chassagnon I.R., McCarthy C.A., Chin Y.K.-Y., Pineda S.S., Keramidas A., Mobli M., Pham V., De Silva T.M., Lynch J.W., Widdop R.E. (2017). Potent Neuroprotection after Stroke Afforded by a Double-Knot Spider-Venom Peptide That Inhibits Acid-Sensing Ion Channel 1a. Proc. Natl. Acad. Sci. USA.

[45] Ryu J.H., Jung H.J., Konishi S., Kim H.H., Park Z.-Y., Kim J.I. (2017). Structure-Activity Relationships of ω-Agatoxin IVA in Lipid Membranes. Biochem. Biophys. Res. Commun..

[46] Qu Y., Liang S., Ding J., Liu X., Zhang R., Gu X. (1997). Proton Nuclear Magnetic Resonance Studies on Huwentoxin-I from the Venom of the Spider Selenocosmia Huwena: 2. Three-Dimensional Structure in Solution. J. Protein. Chem..

[47] Liu Z., Dai J., Dai L., Deng M., Hu Z., Hu W., Liang S. (2006). Function and Solution Structure of Huwentoxin-X, a Specific Blocker of N-Type Calcium Channels, from the Chinese Bird Spider Ornithoctonus Huwena*. J. Biol. Chem..

[48] Kyte J., Doolittle R.F. (1982). A Simple Method for Displaying the Hydropathic Character of a Protein. J. Mol. Biol..

[49] Wang M., Rong M., Xiao Y., Liang S. (2012). The Effects of Huwentoxin-I on the Voltage-Gated Sodium Channels of Rat Hippocampal and Cockroach Dorsal Unpaired Median Neurons. Peptides.

[50] Winterfield J.R., Swartz K.J. (2000). A Hot Spot for the Interaction of Gating Modifier Toxins with Voltage-Dependent Ion Channels. J. Gen. Physiol..

[51] Salari A., Vega B.S., Milescu L.S., Milescu M. (2016). Molecular Interactions between Tarantula Toxins and Low-Voltage-Activated Calcium Channels. Sci. Rep..

[52] Jung H.J., Lee J.Y., Kim S.H., Eu Y.J., Shin S.Y., Milescu M., Swartz K.J., Kim J.I. (2005). Solution Structure and Lipid Membrane Partitioning of VSTx1, an Inhibitor of the KvAP Potassium Channel. Biochemistry.

[53] Ingólfsson H.I., Carpenter T.S., Bhatia H., Bremer P.-T., Marrink S.J., Lightstone F.C. (2017). Computational Lipidomics of the Neuronal Plasma Membrane. Biophys. J..

[54] Logashina Y.A., Mosharova I.V., Korolkova Y.V., Shelukhina I.V., Dyachenko I.A., Palikov V.A., Palikova Y.A., Murashev A.N., Kozlov S.A., Stensvåg K. (2017). Peptide from Sea Anemone Metridium Senile Affects Transient Receptor Potential Ankyrin-Repeat 1 (TRPA1) Function and Produces Analgesic Effect. J. Biol. Chem..

[55] Logashina Y.A., Solstad R.G., Mineev K.S., Korolkova Y.V., Mosharova I.V., Dyachenko I.A., Palikov V.A., Palikova Y.A., Murashev A.N., Arseniev A.S. (2017). New Disulfide-Stabilized Fold Provides Sea Anemone Peptide to Exhibit Both Antimicrobial and TRPA1 Potentiating Properties. Toxins.

[56] Andreev Y.A., Kozlov S.A., Vassilevski A.A., Grishin E.V. (2010). Cyanogen Bromide Cleavage of Proteins in Salt and Buffer Solutions. Anal. Biochem..

[57] Kovalchuk S.I., Jensen O.N., Rogowska-Wrzesinska A. (2019). FlashPack: Fast and Simple Preparation of Ultrahigh-Performance Capillary Columns for LC-MS. Mol. Cell Proteom..

[58] Bax A., Vuister G.W., Grzesiek S., Delaglio F., Wang A.C., Tschudin R., Zhu G. (1994). Measurement of Homo- and Heteronuclear J Couplings from Quantitative J Correlation. Meth. Enzymol..

[59] Kazimierczuk K., Orekhov V.Y. (2011). Accelerated NMR Spectroscopy by Using Compressed Sensing. Angew. Chem. Int. Ed. Engl..

[60] Koradi R., Billeter M., Wüthrich K. (1996). MOLMOL: A Program for Display and Analysis of Macromolecular Structures. J. Mol. Graph..

[61] Cole R., Loria J.P. (2003). FAST-Modelfree: A Program for Rapid Automated Analysis of Solution NMR Spin-Relaxation Data. J. Biomol. NMR.

[62] Bystrov V.F., Dubrovina N.I., Barsukov L.I., Bergelson L.D. (1971). NMR Differentiation of the Internal and External Phospholipid Membrane Surfaces Using Paramagnetic Mn2+ and Eu3+ Ions. Chem. Phys. Lipids.

[63] Shenkarev Z.O., Shulepko M.A., Bychkov M.L., Kulbatskii D.S., Shlepova O.V., Vasilyeva N.A., Andreev-Andrievskiy A.A., Popova A.S., Lagereva E.A., Loktyushov E.V. (2020). Water-Soluble Variant of Human Lynx1 Positively Modulates Synaptic Plasticity and Ameliorates Cognitive Impairment Associated with A7-NAChR Dysfunction. J. Neurochem..

[64] Hu H., Tian J., Zhu Y., Wang C., Xiao R., Herz J.M., Wood J.D., Zhu M.X. (2010). Activation of TRPA1 Channels by Fenamate Nonsteroidal Anti-Inflammatory Drugs. Pflugers Arch..

[65] Raisinghani M., Zhong L., Jeffry J.A., Bishnoi M., Pabbidi R.M., Pimentel F., Cao D.-S., Evans M.S., Premkumar L.S. (2011). Activation Characteristics of Transient Receptor Potential Ankyrin 1 and Its Role in Nociception. Am. J. Physiol. Cell Physiol..

[66] Kneller J.M., Lu M., Bracken C. (2002). An Effective Method for the Discrimination of Motional Anisotropy and Chemical Exchange. J. Am. Chem. Soc..

[67] Pyrkov T.V., Chugunov A.O., Krylov N.A., Nolde D.E., Efremov R.G. (2009). PLATINUM: A Web Tool for Analysis of Hydrophobic/Hydrophilic Organization of Biomolecular Complexes. Bioinformatics.

