# Supplementary material for: Recombinant Production, NMR Solution Structure, and Membrane Interaction of the Phα1β Toxin, a TRPA1 Modulator from the Brazilian Armed Spider Phoneutria nigriventer
Source: Toxins (Basel). 2023 Jun 3;15(6):378. doi: 10.3390/toxins15060378 (PMC10305275; doi:10.3390/toxins15060378)
Supplement: Supplementary file 1 [file toxins-15-00378-s001.zip › toxins-2334228-supplementary.pdf]

Supplementary Materials

Recombinant production, NMR solution structure, and membrane interaction of the Phα1β toxin, a TRPA1 modulator from the Brazilian armed spider *Phoneutria nigriventer*

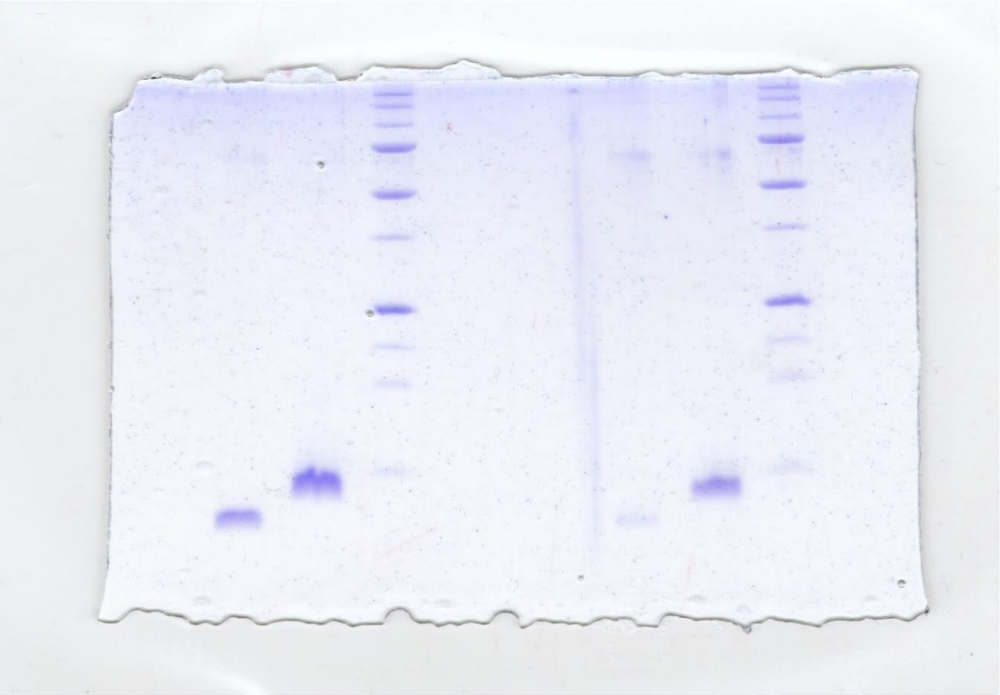

Figure S1. Full SDS-PAGE gel used fort analysis of the purified Phα1β (MW ~ 6 KDa). Agiotoxin-2 (MW ~ 4 KDa) was used as a low molecular weight reference protein. The labels of the lanes and MW markers are shown in Figure 2B in the manuscript. Repeating lines were used to select the optimal concentration of toxins.

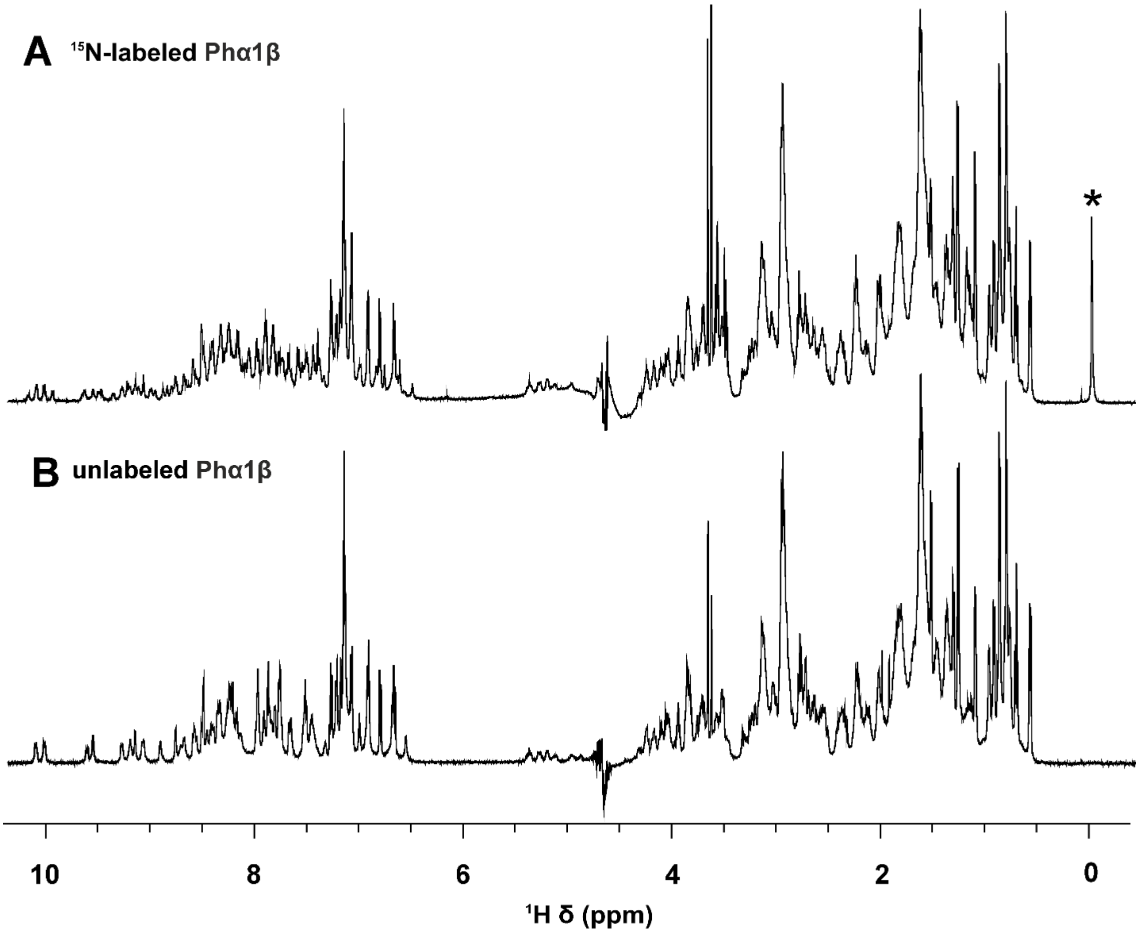

Figure S2. 1D ^{1}H NMR spectra of **(A)** ^{15}N labeled and **(B)** unlabeled recombinant analogues of Phα1β (30 °C, pH 4.5). An impurity containing Si-CH_3, probably originating from the C4 phase of the HPLC column, is marked with an asterisk.

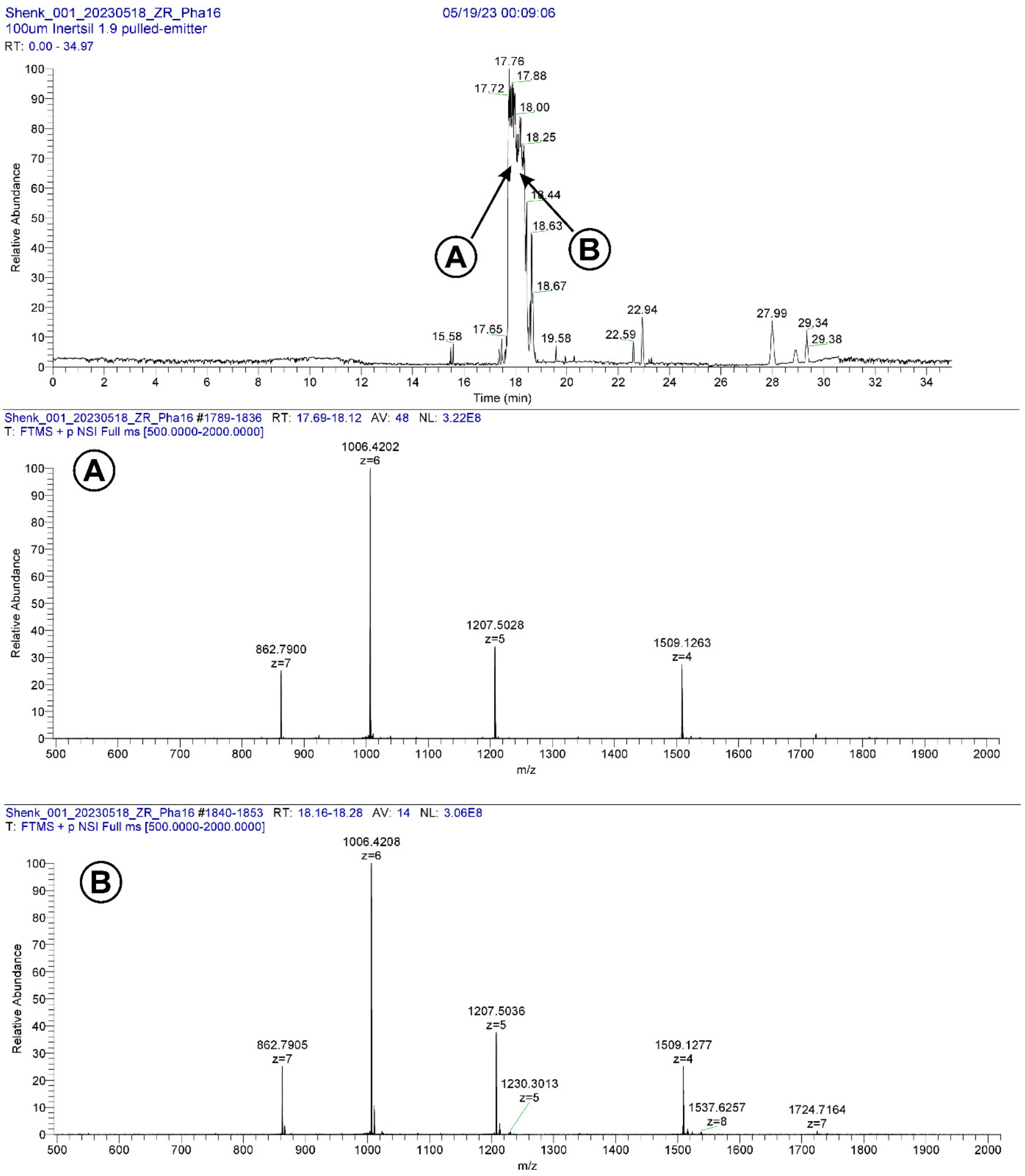

Figure S3. LC-MS analysis of the recombinant Phα1β protein. (top) Reverse-phase (C18) chromatography profile obtained on Ultimate 3000 Nano LC System (Thermo Fisher Scientific). Note, significant overswing in intensity for the two major peaks. (bottom) MS spectra of two major LC peaks measured at Q Exactive Plus Orbitrap mass spectrometer (Thermo Fisher Scientific). Both peaks correspond to the same compound.

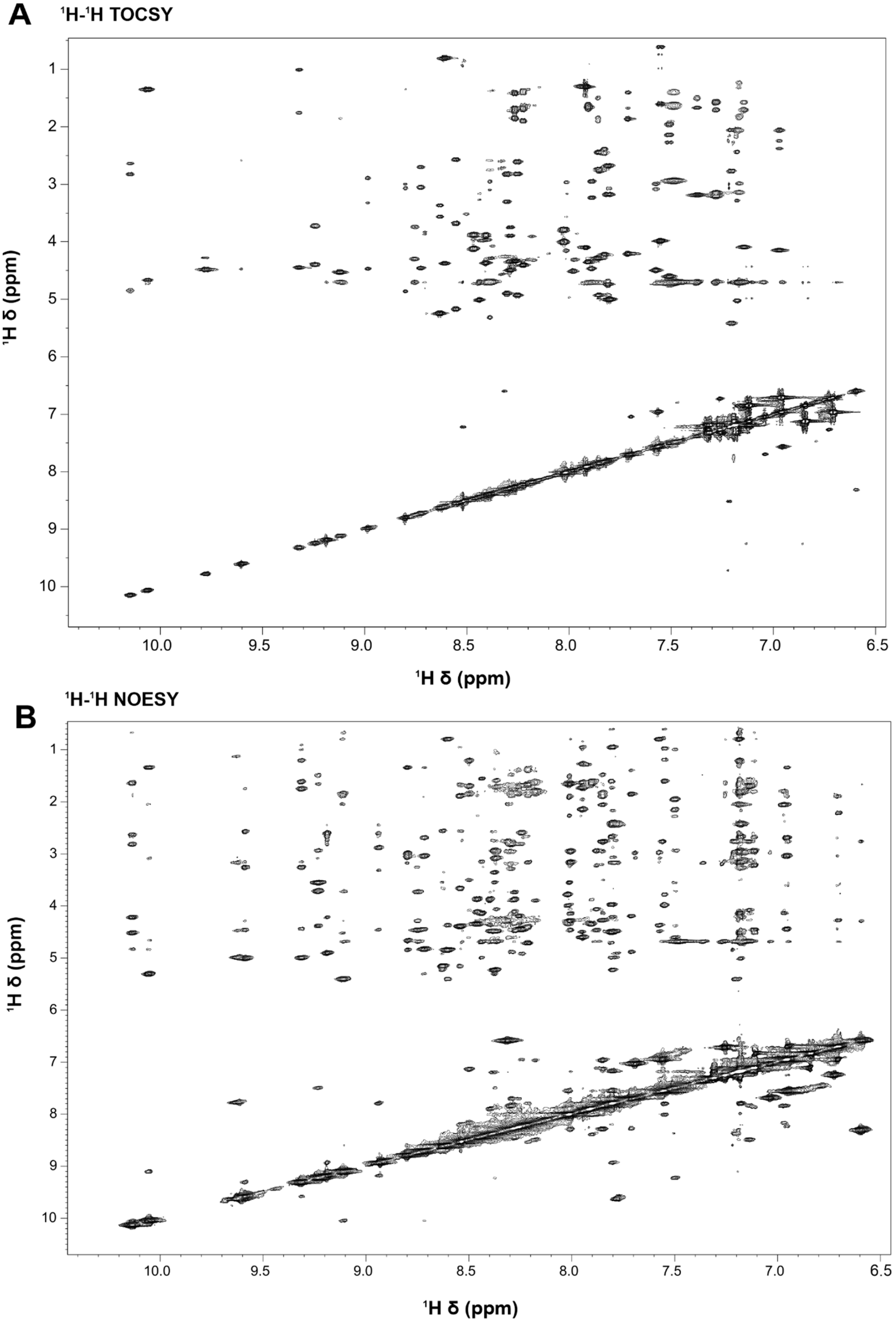

Figure S4. 2D NMR spectra of unlabeled recombinant Phα1β. **(A)** ¹H-¹H TOCSY (τm = 60 ms) and **(B)** ¹H-¹H NOESY (τm = 100 ms) at 30 °C, pH 4.5.

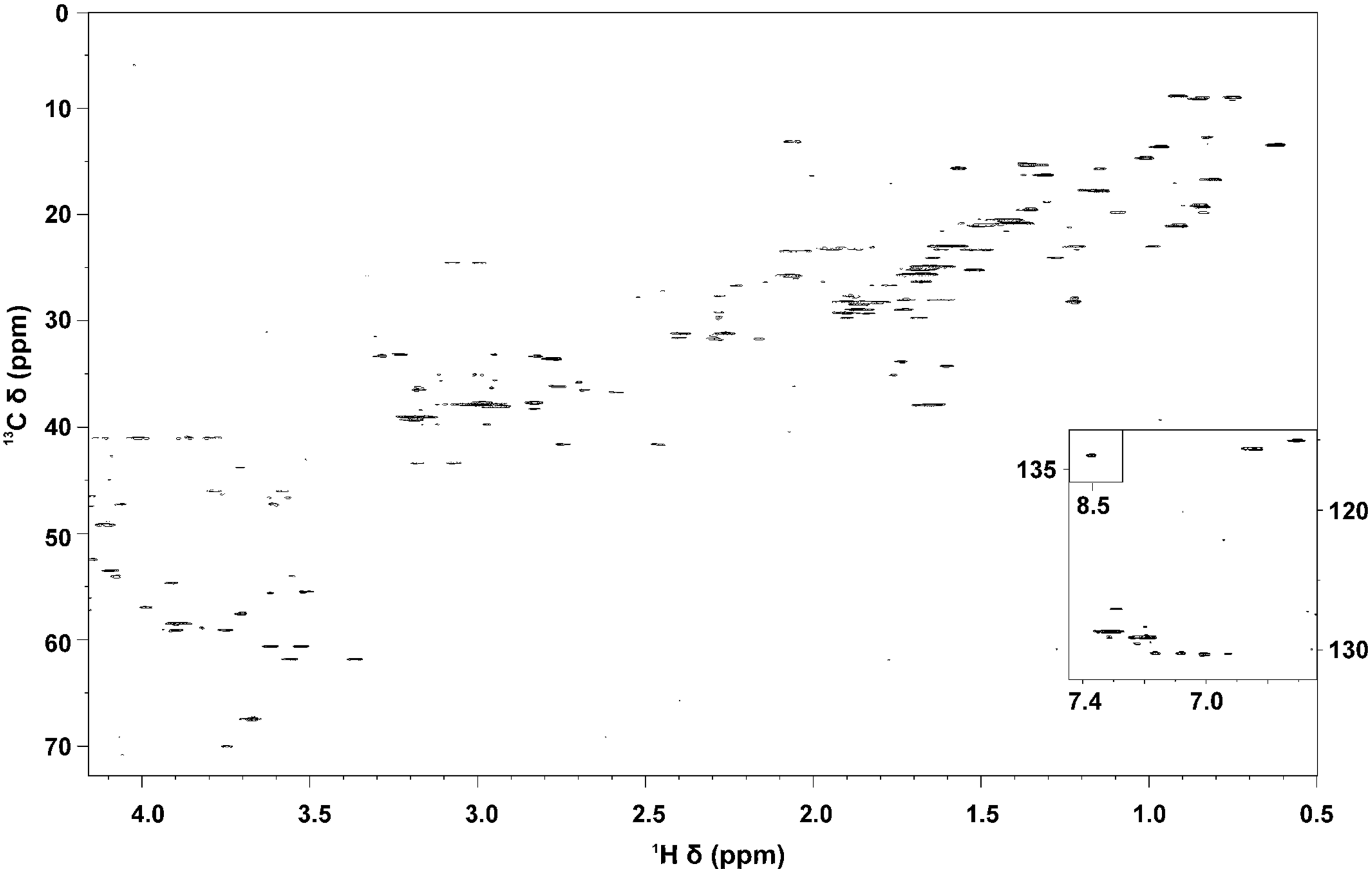

Figure S5. 2D ^{1}H-^{13}C HSQC NMR spectrum of Phα1β (30 °C, pH 4.5).

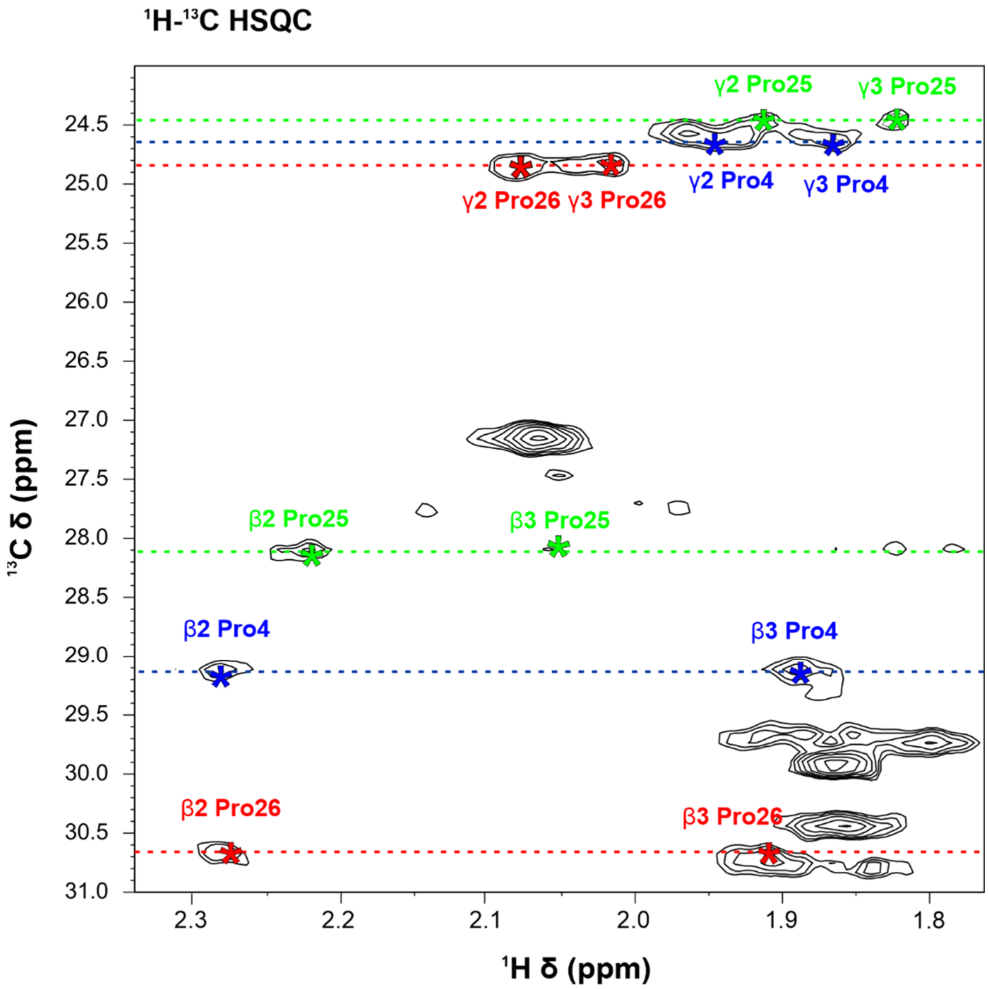

Figure S6. The fragment of 2D ^{1}H-^{13}C HSQC spectrum of Phα1β with HC^{β} and HC^{γ} resonances of prolines (30 °C, pH 4.5). Pro4, Pro25, and Pro26 signals are highlighted by blue, green, and red colors, respectively. The resonance frequencies of C^{β} and C^{γ} signals are marked by dotted lines. The cross-peaks are shown by asterisks and are colored according to the color of the spin system.

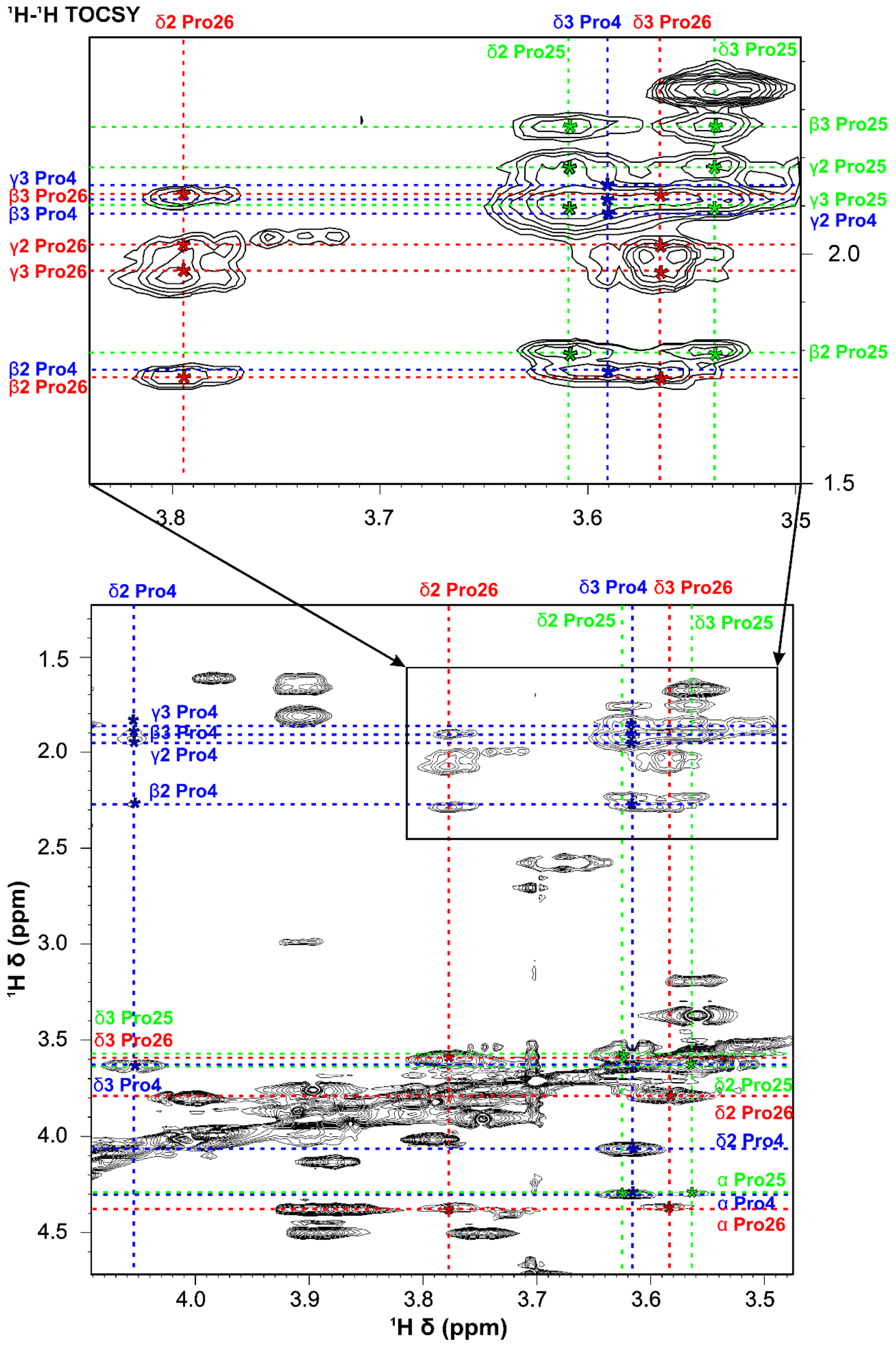

Figure S7. The fragment of 2D ¹H-¹H TOCSY spectrum (τm = 60 ms) of Phα1β with H$^{\delta}$ Pro signals (30 °C, pH 4.5). Pro4, Pro25, and Pro26 signals are highlighted by blue, green, and red colors, respectively. The resonance frequencies of signals are marked by dotted lines. The cross-peaks are shown by asterisks and are colored according to the color of the spin system.

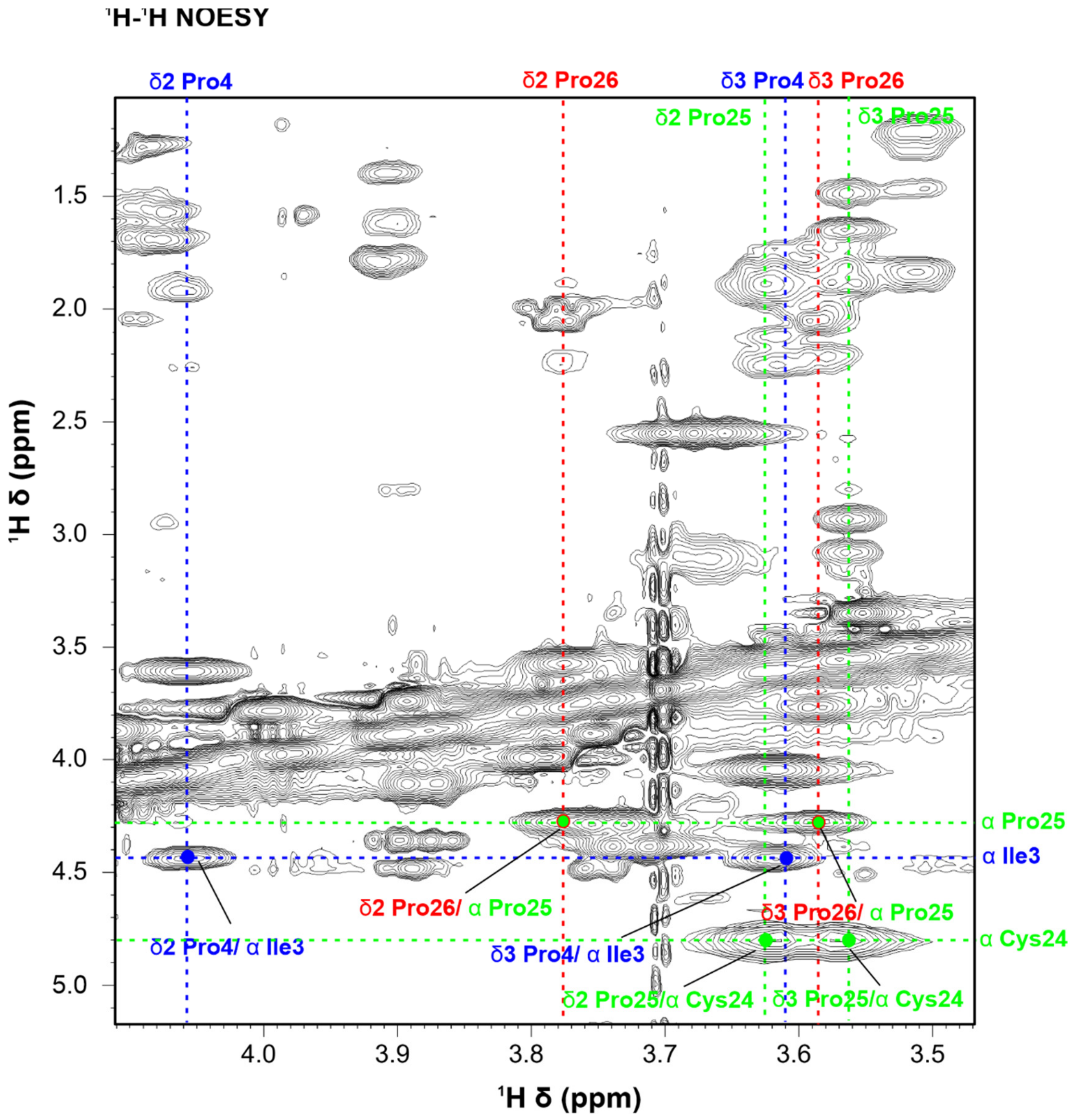

Figure S8. The fragment of 2D ^{1}H-^{1}H NOESY spectrum (τm = 100 ms) of Phα1β with H^{α} - H^{δ} cross-peaks for Xxx-Pro dipeptides (30 °C, pH 4.5). Pro4, Pro25, and Pro26 signals are highlighted by blue, green, and red colors, respectively. The NOE contacts are shown by points and are colored according to the color of the spin system.

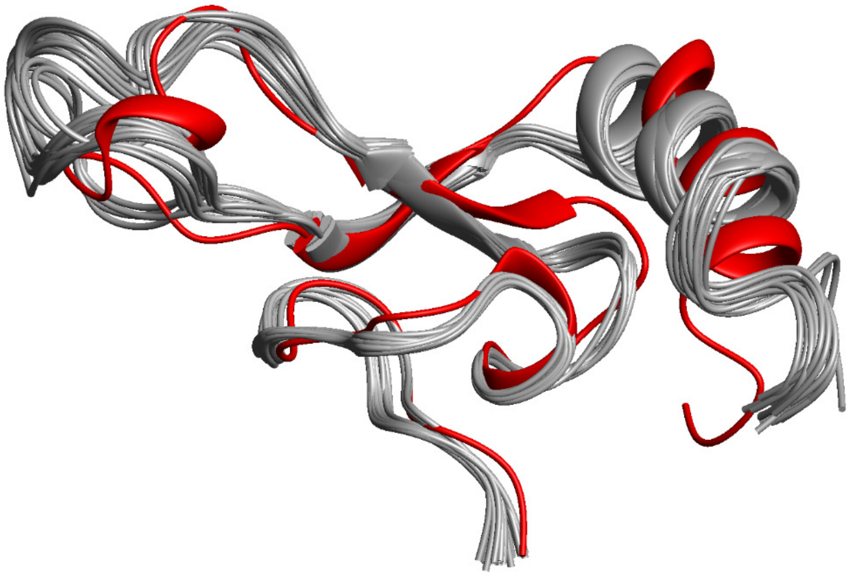

Figure S9. The comparison of experimentally determined Phα1β structure (gray, 20 models) with the model predicted by AlphaFold 2.0 (red). The structures are superimposed by heavy atoms of Cys residues.

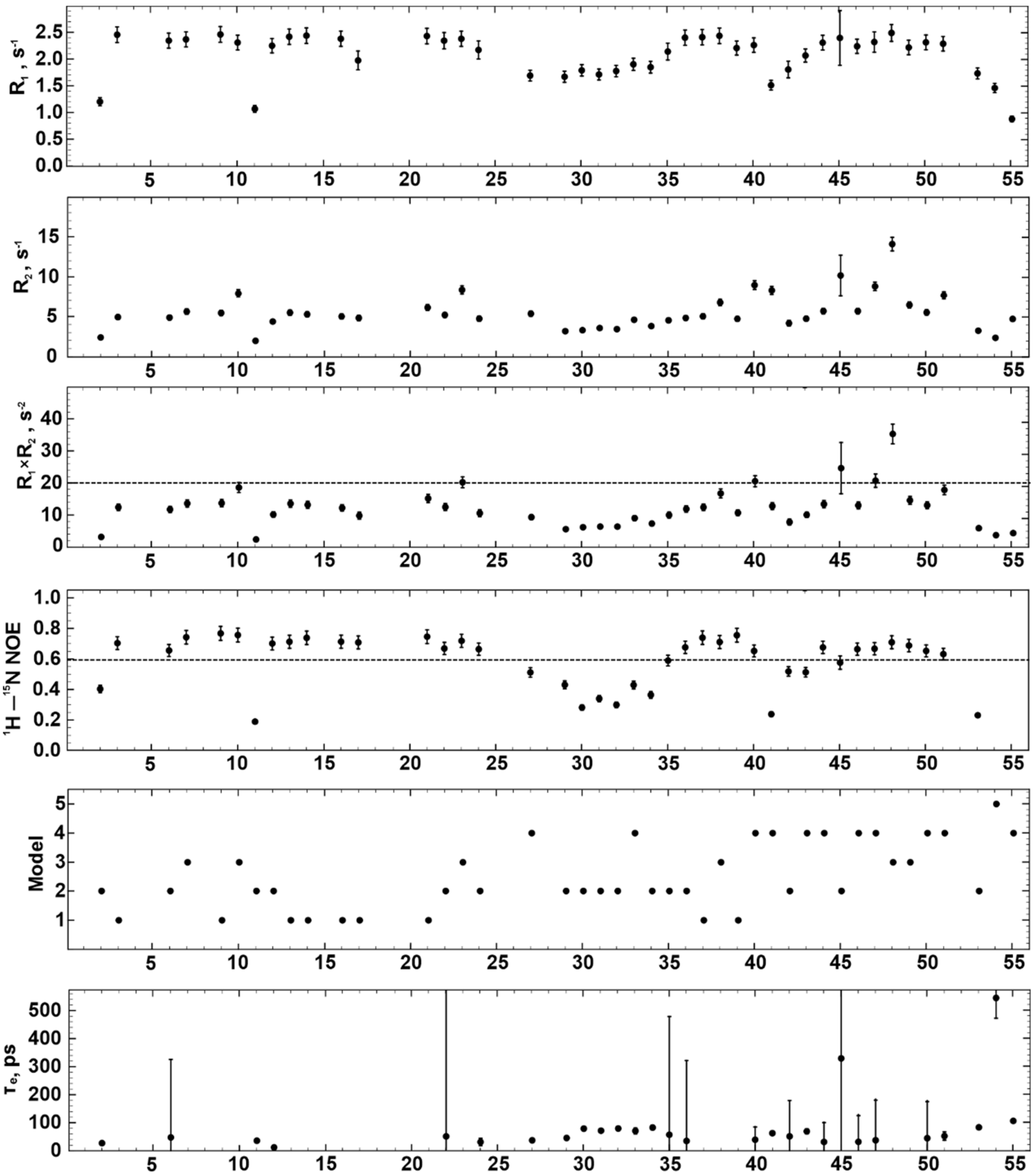

Figure S10. 15N relaxation data for Phα1β (60 MHz, pH 5.3, 30°C) and results of the 'model-free' analysis. R1 and R2 – the values of longitudinal and transverse 15N relaxation rates. 15N-{1H}-NOE - steady-state heteronuclear NOE. Model – the number of relaxation model assigned by the FastModelFree software. τe – effective correlation time for ps and ns backbone motions. The NOE threshold of 0.6 defines the sites of high amplitude ps-ns mobility. Residues displaying R1×R2 > 20 s-2 [66] are subjected to exchange fluctuations in μs-ms timescale.

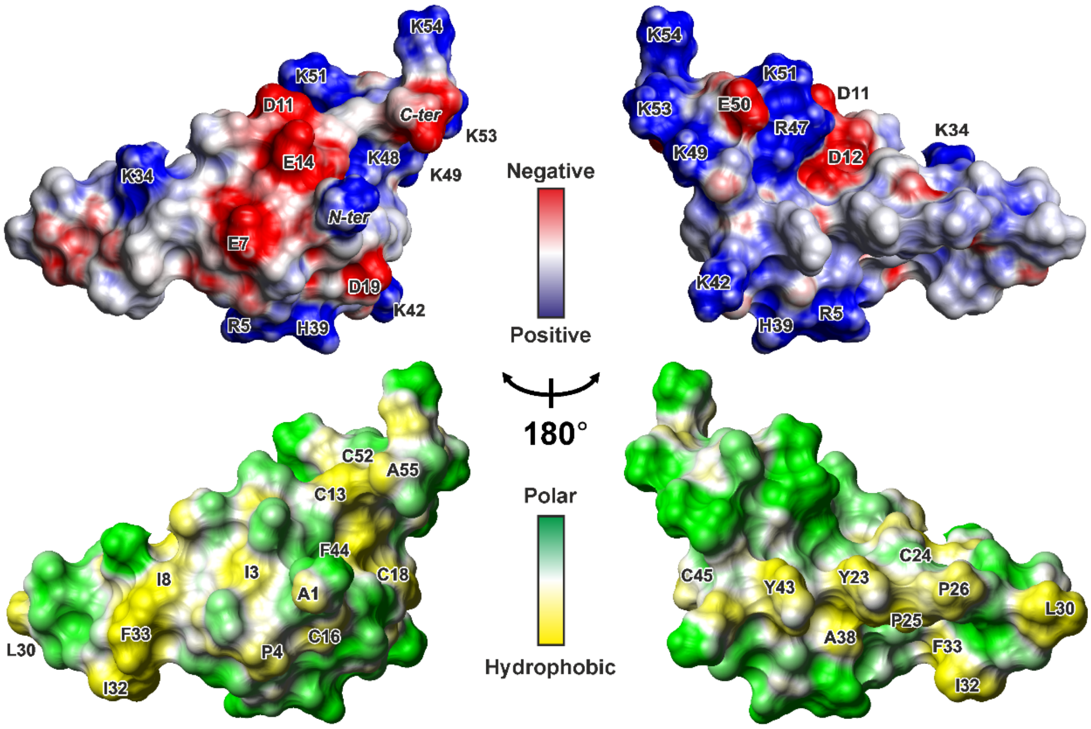

Figure S11. Two-sided view of electrostatic and molecular hydrophobicity [67] potentials on the Phα1β surface. Red, blue, green, and yellow areas denote negative, positive, polar, and hydrophobic regions, respectively.

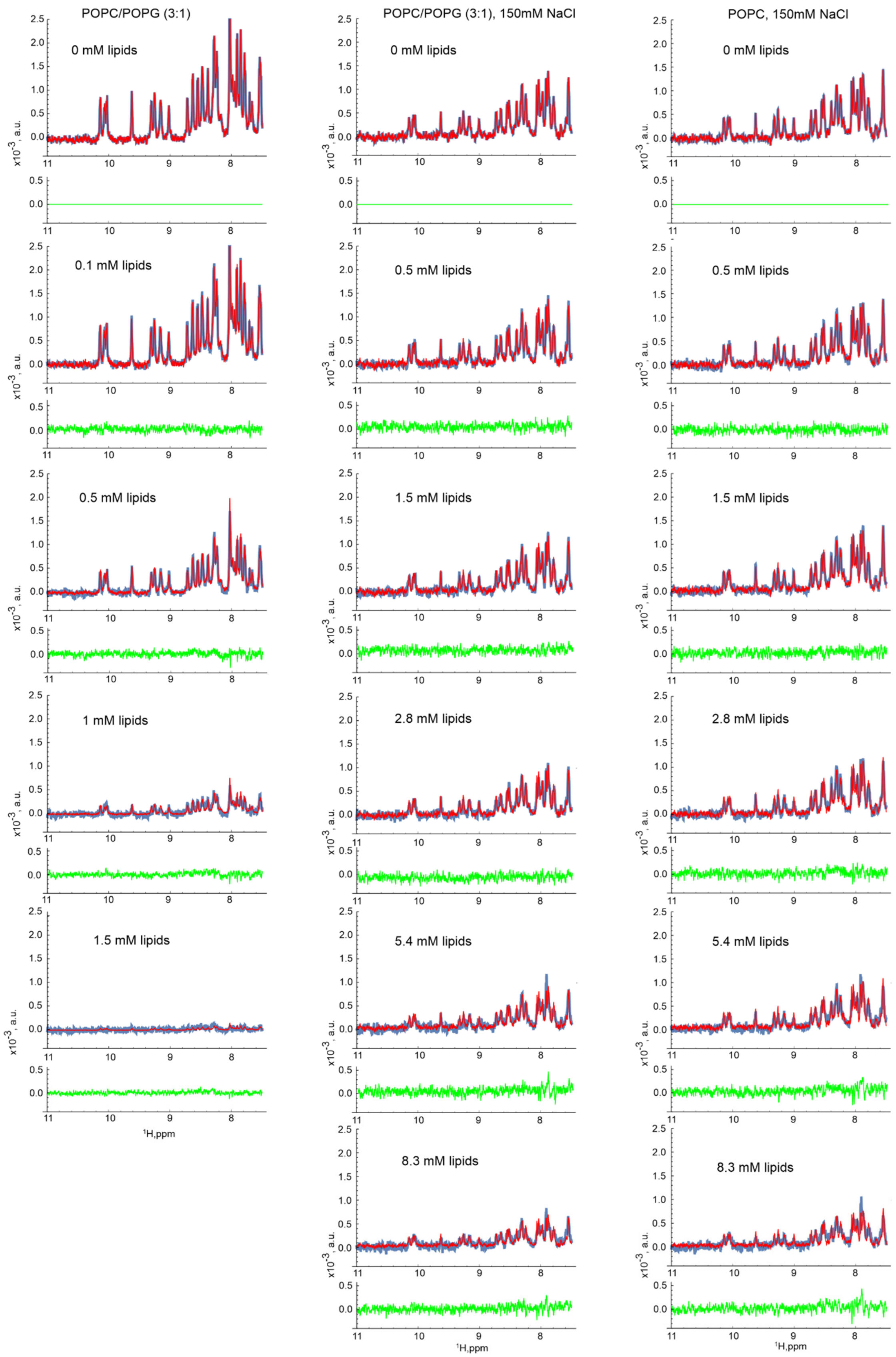

Figure S12. Approximation of the 1D ^{1}H NMR spectra of Phα1β measured in the presence of lipids (blue) by intensity scaled spectrum of the toxin in aqueous solution (red). Sample compositions are shown in the figure above the corresponding columns. The differences between the "blue" and "red" spectra (residuals) are shown by green.

Table S1. Statistics for the best CYANA structures of Phα1β.

**Distance and angle restraints**	
Total NOE contacts	384
Intraresidual	115
Sequential (|i – j| = 1)	170
Medium range (1 < |i – j| < 4)	49
Long range (|i – j| > 4)	53
Hydrogen bond restraints (bonds/upper/lower)	17/34/34
S–S bond restraints (bonds/upper/lower)	6/18/18
Torsion angle restraints	78
Angle φ	43
Angle χ_1	35
Total restraints/per residue	666/12.1
**Statistics for calculated structures**	
Structures calculated/selected	200/20
CYANA target function (Å^2)	1.2± 0.07
Violations of restraints	
Distance (> 0.2 Å)	0
Dihedral angles (> 1°)	0
r.m.s.d. (Å) for stable regions (2-26, 35-54)	
Backbone	0.46 ± 0.19
Heavy atoms	1.02 ± 0.27
r.m.s.d. (Å) overall	
Backbone	0.89 ± 0.37
Heavy atoms	1.46 ± 0.53

r.m.s.d. – root mean square deviation.

Table S2. Residuals after least-squares fit of 1D ^{1}H NMR spectra of Phα1β in the presence of lipids by scaled Phα1β spectrum in water. For each fit, the standard deviation of residual (11-7.5ppm) and standard deviation of noise region (11-10.4ppm) of corresponding spectrum in lipids are shown. ($\times 10^{-5}$, a.u.).

	**POPC:POPG 0mM NaCl**			**POPC:POPG 150mM NaCl**			**POPC 150mM NaCl**		
**# spect.**	**C lipids, x0.6,mM**	**SD Noise**	**SD Fit re-sidual**	**C lipids, x0.6,mM**	**SD Noise**	**SD Fit residual**	**C lipids, x0.6,mM**	**SD Noise**	**SD Fit residual**
1	0.0	3.5	0.0	0.0	4.5	0.0	0.0	3.9	0.0
2	0.1	3.3	5.0	0.5	4.3	6.1	0.5	4.4	5.9
3	0.5	4.1	4.8	1.5	4.0	6.0	1.5	4.5	6.2
4	1.0	3.6	4.4	2.8	4.4	5.9	2.8	4.7	6.4
5	1.5	2.6	3.0	5.4	5.2	7.1	5.4	3.7	7.4
6	-	-	-	8.3	4.7	6.3	8.3	4.5	7.5
